# HNPP: Higher-order network-based personalized PageRank for detecting critical phase in complex biological systems

**DOI:** 10.1371/journal.pcbi.1014475

**Published:** 2026-07-17

**Authors:** Jiayuan Zhong, Xuerong Gu, Dandan Ding, Qiao Wei, Bowen Niu, Ting Tao, Pei Chen, Rui Liu

**Affiliations:** 1 School of Mathematics, Foshan University, Foshan, China; 2 School of Biology and Biological Engineering, South China University of Technology, Guangzhou, China; 3 Department of Nephrology, The Third Affiliated Hospital, School of Medicine, Foshan University, Foshan, China; 4 School of Mathematics, South China University of Technology, Guangzhou, China; University College London, UNITED KINGDOM OF GREAT BRITAIN AND NORTHERN IRELAND

## Abstract

Dynamic biological processes often undergo a critical transition, where the system shifts from one stable state to another with marked qualitative changes. Identifying such a critical state and its associated signaling molecules provides insight into the mechanisms of complex biological processes and allows timely intervention to avert catastrophic outcomes. However, existing critical point detection approaches are predominantly formulated on pairwise interactions, which insufficiently capture the nonlinear and higher-order dependencies inherent in high-dimensional biological data, thereby limiting their robustness and accuracy, especially in single-cell transcriptomic analyses. To address this challenge, we propose a new framework called higher-order network-based personalized PageRank (HNPP) to identify critical phases and signaling molecules at the single-cell level. By incorporating higher-order collaborative structures, HNPP captures many-body interaction patterns that extend beyond traditional pairwise relationships, enabling a more accurate characterization and quantification for the criticality of complex biological systems. The effectiveness of our proposed HNPP has been validated using a simulated dataset and six distinct real-world single-cell datasets. In addition, the results demonstrate that HNPP exhibits enhanced early-warning capability and higher accuracy compared to existing critical point detection methods. Furthermore, the computational findings are reinforced by functional analysis of the identified signaling molecules.

## Author summary

In complex biological processes, such as embryonic development and disease progression, there exists a critical phase or tipping point preceding the transition, where a considerable qualitative shift occurs. Accurate identification of such critical phases and their associated signaling molecules is essential for understanding the underlying mechanisms of biological processes and enabling timely interventions to prevent adverse outcomes. However, existing methods mainly rely on pairwise gene relationships, potentially overlooking higher-order interactions among multiple genes, and often exhibit limited robustness and effectiveness when applied to noisy and sparse single-cell data. To address this challenge, we developed HNPP, a higher-order network-based personalized PageRank framework for detecting critical phases from single-cell data. By incorporating higher-order collaborative structures through simplicial complexes, HNPP captures many-body interaction patterns beyond conventional pairwise relationships, providing a more accurate characterization of critical dynamics in complex biological systems. We validated the proposed method using one simulated dataset and six real-world single-cell datasets spanning embryonic development and disease progression. Our results show that HNPP identifies critical phases more effectively than existing methods, providing a useful tool for studying dynamic biological transitions.

## Background

**Fig 1:**
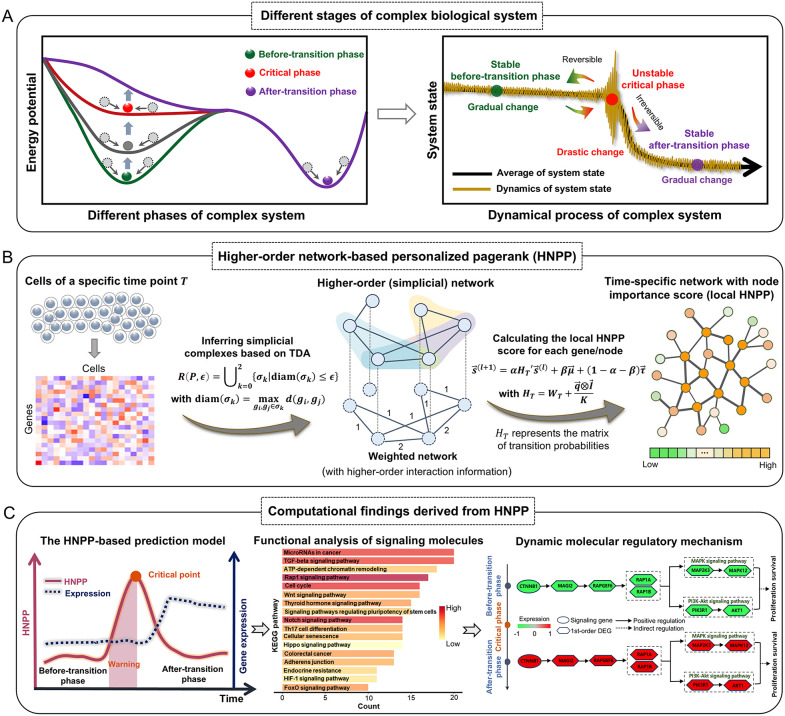
An overview diagram of the HNPP approach used to detect critical phases in complex biological systems. **(A)** In general, the dynamics of complex biological processes progress through three characteristic states: (i) a stable before-transition phase with strong resistance to disturbance; (ii) an unstable and sensitive critical phase or tipping point; and (iii) a re-stabilized after-transition phase. **(B)** The time-specific graph is derived from higher- simplicial networks, followed by calculation of the local HNPP scores for each node or gene using a modified personalized PageRank algorithm. **(C)** HNPP is employed to conduct the following primary analyses: detection of critical phases in complex biological systems, functional analysis of signaling molecules, and exploration of potential molecular regulatory signaling pathways.

Many complex biological systems experience abrupt transitions, characterized by a rapid shift from one stable state to another distinct state [1]. From the viewpoint of dynamical systems, complex biological processes typically evolve over time through three characteristic stages (Fig 1A) [2,3]: (i) a stable before-transition phase with strong resistance to disturbance; (ii) a critical phase or tipping point, where the system becomes unstable and highly sensitive to changes; and (iii) a re-stabilized after-transition phase. The critical phase marks the limit of the reversible before-transition phase, while the resulting catastrophic changes are typically irreversible once the system transitions into the after-transition phase. In recent years, the identification of critical phases for complex biological processes, such as embryonic development or cell differentiation [4,5] and disease progression [6–8], has become an increasingly prominent focus. Accurate identification of such critical phases is essential for uncovering the intrinsic mechanisms of biological processes and for implementing timely interventions to prevent catastrophic events and mitigate their negative consequences. For example, pinpointing the critical state during embryonic development is vital for developing individualized disease models and assessing patient-specific therapeutic responses [9]. The detection of critical phase for complex diseases can help prevent further deterioration and effectively control disease progression [10]. Therefore, it is of great significance to detect the critical phase for complex biological systems. Nevertheless, the precise detection of the critical phase during complex biological processes remains a significant challenge, owing to the similarities in molecular expression and phenotype between the before-transition and critical phases, as well as issues posed by high-dimensional, noisy data, and imperfect predictive models.

Recently, we developed a theoretical framework called the dynamic network biomarker (DNB) [2,11] to pinpoint critical point prior to the state transition of biological systems. Based on various types of biological data, including bulk transcriptomic data and single-cell data, numerous DNB-based methods have been employed in various biological contexts, such as critical state identification during complex diseases progression [12–14], cell-fate transition detection for cell differentiation [15], and studies on immune checkpoint blockade [16]. Single-cell RNA sequencing (scRNA-seq) enables gene expression profiling at the resolution of individual cells, providing unprecedented opportunities to study dynamic cellular processes and the underlying molecular regulatory networks [17,18], In recent years, several relevant frameworks have been developed to model cell dynamics, analyze attractor or cell-state transitions, and infer gene regulatory changes from single-cell transcriptomic data. In particular, spliceJAC uses spliced and unspliced mRNA information from scRNA-seq data to infer cell-state-specific regulatory networks and identify potential driver genes involved in cell-state transitions [19]. MuTrans applies a multiscale reduction framework to characterize the stochastic dynamics of cell-fate transitions and distinguish stable and transition cells [20]. However, in the context of scRNA-seq data analysis, the effectiveness of most traditional DNB approaches may be constrained by substantial transcript-level amplification noise and inherent data sparsity [8,21]. Moreover, these computational approaches rely on correlations between pairs of molecules (node-to-node interactions) within simple network structures, while multi-body interaction patterns beyond pairwise relationships are widely observed in many real complex systems [22]. The higher-order networks have been shown to capture many-body interactions and more effectively reveal the underlying mechanisms driving the dynamics of complex systems [23,24]. Numerous methods based on higher-order structures have been applied to a wide range of biological problems, including epidemic modeling [25], gene–drug regulatory module identification [26], cell clustering [27], pseudotime trajectory inference [28], and cell–cell communication analysis [29]. It is regarded that an appropriate integration of not only the conventional pairwise interaction information but also the higher-order collaborative structures into early-warning frameworks is beneficial to improving the stability and informativeness in detecting critical transitions or bifurcation points within complex systems [24,30]. Thus, there is an urgent need to design novel higher-order network-based approaches specifically adapted to high-dimensional single-cell data, facilitating the accurate detection of critical phase for complex biological systems and the identification of key signaling molecules.

In this research, from the viewpoint of higher-order interactions, we propose a novel and generalized framework called higher-order network-based personalized PageRank (HNPP) to detect the critical phase or critical transition points of complex biological processes from single-cell data. Specifically, simplicial complexes can be constructed using topological data analysis (TDA) (Fig 1B) [31,32], a technique for integrating topology and data analysis to effectively extract structural features from high-dimensional, complex, and nonlinear datasets. As an extension from pairwise to many-body interactions, simplicial complexes provide a unified mathematical framework for modeling higher-order dynamics, capturing not only the combinatorial features but also the underlying topological and geometric properties of higher-order networks. Moreover, the time-specific network or graph is reconstructed based on higher-order (simplicial) structures, after which the local HNPP for each node/gene is computed via a modified personalized PageRank model (Fig 1B). Unlike the traditional DNB algorithm, our proposed method leverages local HNPP score to characterize the network’s critical properties rather than expression-level fluctuations, thereby providing a more reliable quantification of network dynamics. The critical phase or tipping point of complex biological systems can be identified by a marked increase in HNPP score, owing to its ability to capture the dynamic changes in higher-order interactions from simplicial networks. To demonstrate the reliability and effectiveness of HNPP, we conducted verification through numerical simulations and six distinct real-world single-cell datasets, including embryonic developmental processes such as pericyte-to-neuron reprogramming, differentiation of human embryonic stem cells into definitive endoderm cells, the transition from inner cell mass to visceral endoderm cells, and human retinal pigment epithelium development, as well as complex disease-related phenomena like erlotinib resistance in lung cancer and T cell exhaustion in liver cancer. Our results demonstrate that the proposed HNPP effectively identifies critical phases across diverse biological processes and uncover key signaling molecules from single-cell data. Furthermore, it achieves enhanced performance than other critical transition detection methods in capturing critical signals of biological systems. In addition, we further validated the effectiveness of HNPP by performing functional analysis on the signaling molecules (Fig 1C). Overall, we introduce a novel computational approach specifically designed for single-cell data, enabling the dynamic tracking of biological systems within the framework of higher-order network.

## Materials and methods

### Theoretical background

In terms of a dynamical systems, a complex biological process evolves dynamically as high-dimensional nonlinear systems, with marked qualitative shifts interpreted as phase transitions at tipping points [33]. As illustrated in Fig 1A, the system’s dynamic progression comprises three main states: a resilient before-transition phase, an unstable critical phase marked by instability and increased sensitivity to disturbances, and a re-stabilized after-transition state phase. According to DNB theory [2,11], as the system nears a critical point, a key group of molecules called the DNBs appears, characterized by three main characteristic features (See S1 Text for details):

- The variability of each molecule within the DNBs increases sharply;
- The correlations among molecules within the DNBs significantly strengthen;
- The correlations between DNBs and those outside the group weaken.

DNB properties imply that, near the critical point, a group of fluctuation-prone and tightly correlated biomolecules exhibiting strong cooperative associations mark the forthcoming critical transition. Actually, the qualitative state transition of complex biological system can be detected through analyzing how such dominant variables in molecular associations evolve at the network level. Therefore, our proposed HNPP is designed to capture the criticality of biological systems by incorporating the structural information of higher-order (simplicial) networks into a modified personalized PageRank model (Fig 1B), which effectively quantifies quantify dynamic shifts in higher-order interactions to enable more accurate detection.

In this study, we infer higher-order interaction and reconstruct simplicial complexes based on topological data analysis (TDA) [31,32], which offers a powerful approach to uncover higher-order structural characteristics from high-dimensional single-cell datasets. Specifically, the simplicial complex inferred by TDA, widely used to describe the topological structure of biological data, is a combinatorial structure consisting of simplices such as vertices (0-simplex), edges (1-simplex), triangles (2-simplex), and higher-dimensional structures. In particular, the Rips complex is a type of simplicial complex that captures the topological relationships between nodes by connecting those within a specified distance threshold. Given a set of nodes/genes $P={\{g}_{\text{1}},g_{\text{2}},\cdots ,g_{n}\}$ and a distance function *d*, the Rips complex is constructed by considering all pairs and triplets of nodes whose pairwise distances are below a specified threshold ϵ. Formally, the Rips complex R(P,ϵ) is defined as follows: (i) 0-simplices (vertices): each node in *P* is considered a vertex; (ii) 1-simplices (edges): an edge $\left\{g_{i},g_{j}\right\}$ is included if the distance $d{(g}_{i},g_{j})$ between genes $g_{i}$ and $g_{j}$ is less than or equal to ϵ; and (iii) 2-simplices (triangles): a triangle $\left\{g_{i},g_{j},g_{l}\right\}$ is included if the pairwise distances $d{(g}_{i},g_{j})$, $d{(g}_{i},g_{l})$, and $d{(g}_{j},g_{l})$ are all less than or equal to ϵ. Thus, the Rips complex R(P,ϵ) is formed by:


$$\begin{array}{c} R(P,\epsilon )={⋃}_{k=\text{0}}^{\text{2}}\left\{\sigma_{k}|\text{diam}(\sigma_{k})\leq \epsilon \right\}, \end{array}$$ (1)

where $\sigma_{k}$ denotes the *k*-simplex formed by nodes in *P*, and its diameter $\text{diam}\left(\sigma_{k}\right)$ is defined as the maximum pairwise distance among all vertices in the simplex:


$$\begin{array}{c} \text{ diam}\left(\sigma_{k}\right)=\underset{g_{i},g_{j}\in \sigma_{k}}{\text{max}}d(g_{i},g_{j}). \end{array}$$ (2)

Especially, the 2-simplex complex can be expressed by:


$$\begin{array}{c} {R_{\text{2}}(P,\epsilon )=\{\{g_{i},g}_{j},g_{l}\}\left|d\left(g_{i},g_{j}\right)\leq \epsilon ,d(g_{i},g_{l})\leq \epsilon ,d(g_{j},g_{l})\leq \epsilon \right\}, \end{array}$$ (3)

$$\begin{array}{c} \text{diam}\left(\sigma_{\text{2}}\right)=\text{max}\left\{d\left(g_{i},g_{j}\right),d\left(g_{i},g_{l}\right),d\left(g_{j},g_{l}\right)\right\}, \end{array}$$ (4)

where parameter ϵ defines the allowable distance for triplets to form a triangle.

Moreover, the PageRank method can be employed to characterize and quantify changes in molecular cooperative associations at the network level, thereby enabling the assessment of significant variations in higher-order structures. Consider G=(V,E) as a network/graph with *K* nodes, where $A=(a_{ij})\in R^{K\times K}$ denotes its adjacency (transition) matrix and d(i) represents the degree of node *i*. For each node *i* (corresponding to the *i*-th row), two scenarios are considered: (1) the degree d(i)>0, $a_{ij}=\text{1}/d(i)$ if node *i* is connected to node *j* in graph *G*, and 0 otherwise; and (2) when degree d(i)=0, $a_{ij}=\text{1}$ if i=j and 0, otherwise. In the graph *G*, the PageRank vector $\vec{s^{*}}$, which reflects the importance score of each node, is determined by finding the fixed point of the following iterative process [34]:


$$\begin{array}{c} {\vec{s}}^{\left(l+\text{1}\right)}=\alpha A^{\prime }{\vec{s}}^{\left(l\right)}+(\text{1}-\alpha )/K. \end{array}$$ (5)

Here, the damping factor α is usually taken as 0.85, while the symbol “′” indicates the matrix transpose operation. In light of the statistical characteristics inherent to DNB theory, this study introduces a modified personalized PageRank model as described below:


$$\begin{array}{c} {\vec{s}}^{\left(l+\text{1}\right)}=\alpha {H_{T}}^{\prime }{\vec{s}}^{\left(l\right)}+\beta \vec{\mu }+(\text{1}-\alpha -\beta )\vec{\tau }, \end{array}$$ (6)

where $H_{T}=W_{T}+\frac{\vec{q}⨂\vec{I}}{K}$ (with ⨂ denoting the outer product operation), the matrix $W_{T}$ (as detailed in Step 2 of the following HNPP method) represents the personalized transition matrix constructed from the time-specific network at time point *T*, $\vec{q}$ is a *K*-dimensional column vector compensating for errors introduced by isolated nodes (see Step 3 of the HNPP method), and $\vec{I}$ denotes a *K*-dimensional row vector of all ones. The TF-based vector $\vec{\mu }$, constructed based on transcription factors (TF), serves to highlight the significance of TF-associated nodes in the network, with its weighting coefficient 𝛽 set to 0.05. The vector $\vec{\tau }$ acts as the personalized vector, as indicated in Step 3 of the HNPP method. To disentangle the effects of the major components of HNPP, we compared three settings: a pairwise DNB-based method without simplicial structures, an HNPP variant retaining the simplicial component but excluding the personalized PageRank modification (i.e., without the TF-based vector $\vec{\mu }$), and the full HNPP model as proposed (S1 Fig and S2 Text). Our findings suggest that the higher-order simplicial construction makes an independent contribution to the observed performance gains beyond those achieved by standard pairwise DNB-based analysis, while personalized PageRank modification with the TF-based vector $\vec{\mu }$ offer further complementary improvements. Overall, the improved performance of HNPP arises from the joint contribution of these components, with the higher-order structure playing a central role in the improvement.

### HNPP method designed for critical phase detection in complex biological process

In the context of a biological system with *K* variables/genes, the proposed HNPP method is applied to pinpoint critical phase or state transitions in complex biological processes, with its detailed procedure outlined below.

[Step 1] Constructing a time-specific higher-order (simplicial) network/structure ${HN}_{T}$. We implemented the process of constructing simplicial complexes following the criteria set out in Equations (1) and (2), where the pairwise distance is defined as follows:

$$\begin{array}{c} d_{T}\left(g_{i},g_{j}\right)=\text{1}-{\text{PCC}}_{T}(g_{i},g_{j}). \end{array}$$ (7)

Here, ${\text{PCC}}_{T}(g_{i},g_{j})$ represents the Pearson correlation coefficient (PCC) of expression value of genes $g_{i}$ and $g_{j}$at the given time point *T*. It is evident that the distance function $d_{T}\left(g_{i},g_{j}\right)$ is negatively correlated with ${\text{PCC}}_{T}(g_{i},g_{j})$; that is, the higher the Pearson correlation coefficient ${\text{ PCC}}_{T}$, the smaller the distance $d_{T}$, which is consistent with the statistical properties of DNB used in network or graph construction. Moreover, we determined whether each set of three genes (gene triplets) can be connected into a triangle (2-simplex) based on Equations (3) and (7), along with the adjustable parameter 𝜖 mainly set at 0.2, thereby constructing the time-specific higher-order (simplicial) structure ${HN}_{T}$ at time point *T*. Our analysis shows that the parameter 𝜖 within a specific range has little effect on the general pattern of the signal curve (S2 Fig and S3 Text). Although TDA is often used to characterize complex geometric or topological structure, in our framework the underlying metric space is constructed from Pearson-correlation-based gene associations. As a result, our HNPP implementation primarily captures higher-order structural information from a Pearson-derived association space based on pairwise linear correlations, which may limit its ability to recover genuinely nonlinear dependencies. Thus, in our research, nonlinear primarily reflects the higher-order geometric and topological structure represented by simplicial complexes, rather than the explicit modeling of arbitrary nonlinear gene–gene relationships.

[Step 2] Constructing the network’s transition matrix $W_{T}^{HN}$ based on the time-specific higher-order (simplicial) structure ${HN}_{T}$. Specifically, the transition matrix $W_{T}^{HN}={\left(w_{i,j}\right)}_{K\times K}$ is derived from the higher-order simplicial structure ${HN}_{T}$, where the matrix element $w_{i,j}$ is given by:

$$\begin{array}{c} w_{i,j}=\frac{n_{i,j}}{\sum_{j=\text{1}}^{K}n_{i,j}}\text{ if }\sum_{j=\text{1}}^{K}n_{i,j}\neq \text{0}, \end{array}$$ (8)

or


$$\begin{array}{c} w_{i,j}=\left\{ \begin{array}{c} @l\text{0}\;(i\neq j)\\ \text{1}(i=j) \end{array}\right.\text{ if }\sum_{j=\text{1}}^{K}n_{i,j}=\text{0}. \end{array}$$ (9)

Here, $n_{i,j}$ refers to the number of triangles (2-simplices) jointly constructed by the gene pair $g_{i}$ and$g_{j}$ with the remaining K−2 genes.

[Step 3] Building the *K*-dimensional vectors $\vec{q}=(q_{\text{1}},\ldots ,q_{K})$ (isolated-node adjustment), $\vec{\mu }=(\mu_{\text{1}},\ldots ,\mu_{K})$(TF-based information) and $\vec{\tau }=(\tau_{\text{1}},\ldots ,\tau_{K})$ (personalization), respectively. More precisely, the isolated-node adjustment vector $\vec{q}$ designed to compensate for errors introduced by isolated nodes. The *i*-th element/row of the normalized vector $\vec{q}$ is defined as:

$$\begin{array}{c} {\hat{q}}_{i}=\left\{ \begin{array}{c} @l\frac{\text{1}}{\sum_{m=\text{1}}^{K}q_{m}},\sum_{j=\text{1}}^{K}w_{i,j}=\text{0}\\ \;\text{0},\;\sum_{j=\text{1}}^{K}w_{i,j}\neq \text{0} \end{array}\right.\;\;\;, \end{array}$$ (10)

where $w_{i,j}$ represents the weight between nodes *i* and *j*.

The TF-based vector $\vec{\mu }$ assigns a value of 1 to elements corresponding to transcription factors and 0 to all others, thereby emphasizing the regulatory importance of TF-associated nodes. The normalized vector $\vec{\mu }$ is obtained by normalizing each element as:


$$\begin{array}{c} {\hat{\mu }}_{i}=\frac{\mu_{i}}{\sum_{m=\text{1}}^{K}\mu_{m}},i=\text{1},\text{2},\cdots ,K. \end{array}$$ (11)

The personalized vector $\vec{\tau }$ reflects the variability of each gene/node $g_{i}$ by calculating the standard deviation of its expression levels across cells at the sampling time point *T*. Each element of the normalized vector $\vec{\tau }$ is calculated as:


$$\begin{array}{c} {\hat{\tau }}_{i}=\frac{\tau_{i}}{\sum_{m=\text{1}}^{K}\tau_{m}},i=\text{1},\text{2},\cdots ,K. \end{array}$$ (12)

[Step 4] Calculating the PageRank vector ${\vec{s}}^{*}$ (composed of the importance scores/local HNPP assigned to each node). As presented as in Equation (6), the local HNPP (gene-specific local HNPP) can be calculated for each gene/node based on the network’s transition matrix $W_{T}^{HN}$, the isolated-node adjustment vector $\vec{q}$, the TF-based vector $\vec{\mu }$, and the personalized vector $\vec{\tau }$, as obtained above. Moreover, the HNPP at the given time point *T* is computed as follows:

$$\begin{array}{c} \text{HNPP}(T)=\frac{\text{1}}{L}\sum_{i=\text{1}}^{L}\left(s_{i}^{*}/\text{mean}({\vec{s}}^{*})\right), \end{array}$$ (13)

where the adjustable parameters *L* represents the count of genes falling within the top 5% in terms of local HNPP, while $\text{mean}\left({\vec{s}}^{*}\right)$ refers to the average of the PageRank score vector ${\vec{s}}^{*}$. Our results indicate parameter *L* within this range (typically from the top 3% to 10%) do not alter the overall trend of the signal curve, thereby demonstrating the robustness of HNPP to the choice of parameter *L* (S3 Fig). Near the critical state, DNB molecules exhibit collective variations in network-level associations, leading to significant dynamic changes in higher-order structures. Such shifts ultimately yield a marked increase in HNPP(T).

[Step 5] Assessing the critical phase via a one-sample t-test. To examine the capability of HNPP in capturing critical signals, we use one-sample t-test to evaluate whether the critical phase differs significantly from the before-transition phase. The statistic *ST*, defined in Equation (14), measures the significance of the deviation of a constant *z* from the mean of the *n*-dimensional vector $\vec{Z}=(z_{\text{1}},z_{\text{2}},\cdots ,z_{n})$.

$$\begin{array}{c} ST=\sqrt{n}\frac{\text{mean}(\vec{Z})-z}{\text{SD}(\vec{Z})}. \end{array}$$ (14)

Here, $\text{mean}(\vec{Z})$ and $\text{SD}(\vec{Z})$ denote its average value and standard deviation, respectively. The p-value derived from *ST* assesses how significantly *z* deviates from the average of $\vec{Z}$. A p-value smaller than 0.05 (p−value≤0.05) indicates a statistically significant difference, whereas a p-value greater than 0.05 (p−value>0.05) does not. The HNPP(T) index indicates a critical state once it meets two criteria: (*i*) HNPP(T) > HNPP(T−1); and (*ii*) HNPP(T) presents a statistical deviation from prior values (p-value ≤ 0.05), as detailed in S4 Text.

### Overview of data and functional analysis

To evaluate the performance of the HNPP method, it was applied to both numerical simulations and six diverse real-world single-cell datasets: including embryonic developmental processes such as pericyte-to-neuron reprogramming [35] (GEO: GSE113036), differentiation of human embryonic stem cells (hESC) into definitive endoderm cells (DEC) [36] (GEO: GSE75748), the transition from inner cell mass (ICM) to visceral endoderm cells (VEC) [37] (GEO: GSE100597), and human retinal pigment epithelium (HRPE) development [38] (GEO: GSE107618), as well as complex disease-related phenomena like erlotinib resistance in lung cancer [39] (GEO: GSE149383) and the progression from hepatitis to liver cancer [40] (PMID: 36221095). The selection of real-world datasets was based on two criteria. First, all of them contain time-course information or dynamic cellular trajectories that describe complete biological processes. Second, they provide the essential information needed to identify key landmarks of these processes, such as key transitions from experimental observations. All scRNA-seq datasets were preprocessed prior to HNPP analysis, including log-transformation (i.e., log(x+1)) and subsequent gene selection based on zero-expression filtering criteria. Genes with zero expression in more than 50% of cells were removed, which is a commonly adopted practice in scRNA-seq preprocessing to retain informative genes and reduce noise [41]. Detailed information on these datasets can be found in S5 Text. We carried out functional enrichment analyses based on the Metascape [42] and the ClusterProfiler [43], with pathway annotations sourced from the KEGG database.

## Results

### HNPP validation based on simulation data

We applied the HNPP approach to an 8-node synthetic network (S4 Fig), described by stochastic differential equations (Eq. S4), to showcase its performance and capability in identifying early-warning indicators near critical transitions. The dynamic behavior of gene regulatory systems is often modeled using Michaelis–Menten or Hill equation-based frameworks [44,45], which have been used to depict complex biological processes such as transcription [46], cyclic biochemical reactions [47], and various other regulatory functions [48]. The system experiences a critical transition controlled by the parameter *p*, where p=0 marks the bifurcation point. Additional details regarding the dynamical system are provided in S6 Text. Numerical simulations were carried out by sweeping the parameter *p* between −0.5 and 0.2 to demonstrate the HNPP method’s effectiveness in detecting the critical transition of system approaching its bifurcation point.

**Fig 2:**
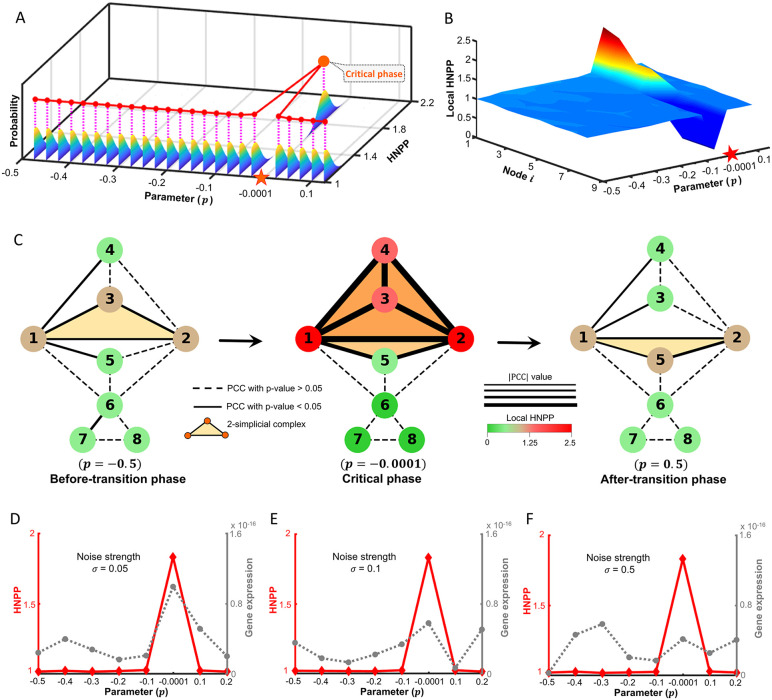
Verification of the HNPP method through the numerical simulation. **(A)** The average HNPP values from simulated trials indicates a marked increase in the vicinity of the bifurcation point (p=0). **(B)** The node-specific patterns of local HNPP are presented, revealing that DNB members undergo a rapid increase in HNPP score as the system transitions toward the bifurcation point. **(C)** Close to the bifurcation point, the regulatory network shows substantial reconfiguration of DNB subnetworks, with every trio of DNBs constituting a 2-simplicial complex. **(D)**-**(F)** A performance comparison between HNPP and molecular expression demonstrates that HNPP exhibits greater robustness and efficacy in pinpointing critical states.

A distinct increase in the HNPP score is observed near a certain parameter value p=0 (Fig 2A), indicating the onset of a critical phase. In addition, the mean HNPP values (red curve in Fig 2A) continue to provide a clear signal of the impending critical point, further demonstrating the robustness of our proposed approach. To clearly illustrate the distinction between the normal phase and the critical phase, Fig 2B shows the overall pattern of local HNPP across different nodes. Local HNPP scores remain consistently low across all nodes when the system is not near a critical point. In contrast, approaching the critical state, a subset of signaling molecules, identified as DNBs, show a sharp rise in their scores. Furthermore, as the system is close to the tipping point, the dynamical evolution of the regulatory network reveals significant reorganization of DNB subnetworks where every trio of DNBs forms a 2-simplicial complex (Fig 2C), signalling an imminent shift in the higher-order structure. Besides, as shown in Fig 2D–2F, a comparison between HNPP and molecular expression was carried out under different noise levels to demonstrate the robustness of the proposed method. With higher levels of noise, our HNPP maintained strong performance and effectively identified critical signals. The findings from the numerical experiments confirm that HNPP effectively captures early indicators of state transitions or critical phases.

### Evaluation of HNPP for critical phase detection in different biological processes

To evaluate the applicability of our proposed method, HNPP was utilized to six distinct scRNA-seq datasets of different biological processes, including pericyte-to-neuron reprogramming [35], hESC-to-DEC differentiation [36], ICM-to-VEC transition [37], human retinal pigment epithelium (hRPE) development [38], lung cancer cells erlotinib-resistance (LCCER) [39], and hepatitis-to-liver-cancer (HELC) progression [37]. Time-specific HNPP scores (as defined in Equation (13)) was applied to detect potential critical phase. The successful detection of critical states across various biological processes demonstrates the accuracy and robustness of HNPP.

**Fig 3:**
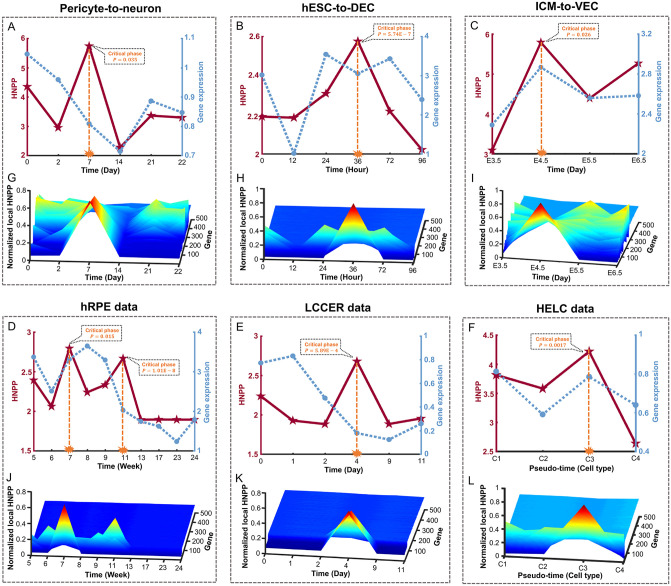
Critical phases of various biological processes revealed by the proposed HNPP. Comparison of dynamic changes between HNPP (rose-red curve) and mean gene expression (light-blue curve) across six real-world single-cell datasets: **(A)** Pericyte-to-neuron, **(B)** hESC-to-DEC, **(C)** ICM-to-VEC, **(D)** hRPE, **(E)** LCCER and **(F)** HELC. Dynamic patterns of signaling and non-signaling genes are illustrated through local HNPP landscape for these datasets: **(G)** Pericyte-to-neuron, **(H)** hESC-to-DEC, **(I)** ICM-to-VEC, **(J)** hRPE, **(K)** LCCER and **(L)** HELC.

For the pericyte-to-neuron data, as shown by the rose-red curve in Fig 3A, a notable increase in the mean HNPP score is observed on day 7 (P=0.035), before the lineage bifurcation into *DLX*- and *NEUROG2*-dominated neuronal fates at day 14 [35]. The hESC-to-DEC data (the rose-red curve of Fig 3B) exhibit a sharp HNPP transition from 24 h to 36 h (P= 5.74E−7), which indicates that definitive endoderm fate commitment occurred at 72h [36]. In ICM-to-VEC data, the rose-red curve in Fig 2C shows a statistically significant shift at embryonic day 4.5 (E4.5) (P=0.026), preceding the epiblast-to-primitive streak transition at E6.5 [37]. For hRPE data, two critical states are detected by HNPP (Fig 3D): the first tipping point at 7 w (P=0.015) precedes the transition around 9 w associated with early retinal pigment epithelium (RPE) differentiation, while the second at 11 w (P=1.01E−8) serves as an indicator of the onset of visual-cycle-related functional maturation and metabolic reprogramming around 13 w [38]. When applied to disease-related LCCER data, it is seen from the rose-red curve of Fig 3E that a pronounced rise (P=5.89E−4) in the average HNPP appears at day 4, marking a critical transition that precedes the emergence of the resistant state in erlotinib-treated PC9 cells at day 9 [39]. For the non-time-series single-cell dataset of HELC, the progression from hepatitis to liver cancer can be partitioned into four distinct clusters via the pseudo-temporal trajectory analysis (see S5 Fig and S7 Text). Fig 3F illustrates a sharp rise (P=0.0017) in the average HNPP score at cluster 3 (C3), indicating a critical shift toward the liver cancer state at C4 [40]. In contrast, the light-blue curves in Fig 3A–3F illustrate dynamic changes in the mean expression levels of the top 5% differentially expressed genes (DEGs), yet such variations were insufficient to offer a reliable indicator of the critical transition. Moreover, the landscape of gene-specific local HNPP was employed to illustrate overall dynamic changes in signaling and non-signaling genes (Fig 3G–3L), revealing sharp elevation in local HNPP for signaling genes. In addition, compared with existing approaches, including Gaussian graphical optimal transport (GGOT) [7], single-sample landscape entropy (SLE) [49], BioTIP [50], directed-network rank score (DNRS) [2], and module-based dynamic network biomarker (M-DNB) [4] (See S8 Text for details), HNPP detects more effective critical signals and provides earlier warning signals before the known biological transition (Table 1), thereby demonstrating its enhanced ability to pinpoint critical phases in complex biological processes. Sensitivity analyses across different cell numbers and data sparsity settings further support the robustness of HNPP (S6 and S7 Figs).

**Table 1:** Comparison of the performance among different critical phase detection methods.

Dataset	Known transition	HNPP	GGOT	SLE	BioTIP	DNRS	M-DNB
Pericyte-to-neuron	14 d	7 d (+1) (P=0.035)	7 d (+1) (P=0.078)	14 d (0) (P=0.064)	14 d (0) (P=7.53E-10)	7 d (+1) (P=4.51E-4)	14 d (0) (P=9.43E-4)
hESC-to-DEC	72 h	36 h (+1) (P=5.74E-7)	None	72 h (0) (P=7.53E-04)	72 h (0) (P=3.48E-7)	36 h (+1) (P=0.0066)	12 h (+3) (P=0.0012) 36 h (+1) (P=0.011)
ICM-to-VEC	E6.5	E4.5 (+2) (P=0.026)	E6.5 (0) (P=4.95E-69)	None	E6.5 (0) (P=0.062)	E6.5 (0) (P= 5.17E-49)	E5.5 (+1) (P=1.39E-7)
hRPE data	9 w 13 w	7 w (+2) (P=0.015) 11 w (+1) (P=1.01E-8)	11 w (-1) (P=5.84E-9) 17 w (-2) (P=8.36E-4)	8 w (+1) (P=0.001) 11 w (+1) (P=1.33E-4)	7 w (+2) (P=0.067)	None	None
LCCER data	9 d	4 d (+1) (P=5.89E-4)	None	None	None	11 d (-1) (P=0.002)	11 d (-1) (P=1.72E-10)
HELC data	C4	C3 (+1) (P=0.0017)	C4 (0) (P=4.25E-5)	C3 (+1) (P=2.27E-5)	C4 (0) (P=0.018)	None	C4 (0) (P=3.74E-5)

* Note: Values in parentheses indicate how early or late each method detects the critical phase relative to the known transition. + 1, + 2, and +3 indicate detection 1, 2, and 3 time points earlier, respectively; 0 indicates detection at the known transition; −1 indicates detection 1 time point later; and None indicates that no critical signal was detected.

### Analysis of the evolving dynamics of signaling genes

**Fig 4:**
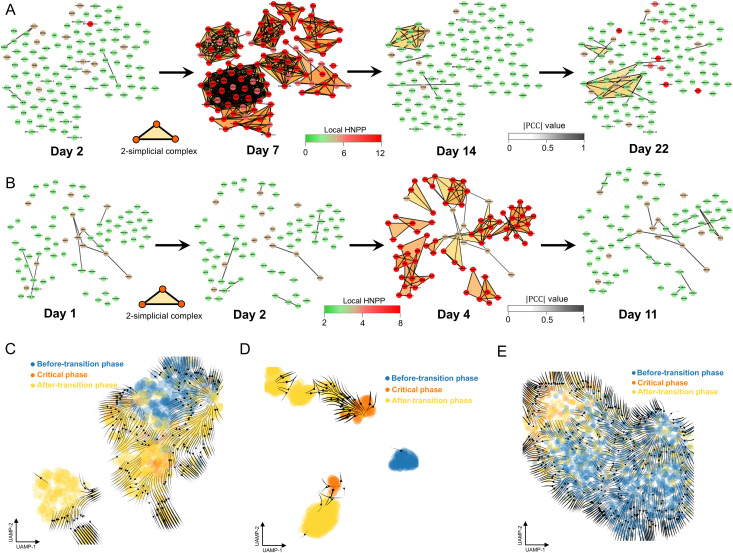
Dynamic evolution analysis of signaling genes. Network dynamics of signaling genes are presented for **(A)** pericyte-to-neuron and **(B)** LCCER. it can be seen that there occurs a notable shift in the network structure on critical state, where every trio of the most prominent signaling genes forms the higher-order structures (2-simplicial complex). Based on the expression profiles of values of the top high- and low-HNPP genes, RNA velocity fields of the before-transition, critical, and post-transition phases are presented for **(C)** pericyte-to-neuron, **(D)** ICM-to-VEC, and **(E)** LCCER. The inferred streamline flows consistently reveal the progression from the before-transition toward the after-transition phase.

At the identified critical phase, we chose the top 5% of genes exhibiting the highest local HNPP values as potential signaling genes, to further explore their roles in the evolving dynamics of biological processes. Specifically, we illustrated the evolution of signaling genes, focusing on their dynamic changes at the network level and how these alterations contribute to the emergence of critical states in complex biological systems. For pericyte-to-neuron data, it can be seen from Fig 4A that there occurs a notable shift in the network structure on day 7, where every trio of the most prominent signaling genes forms the higher-order structures (2-simplicial complex), indicating the cell fate determination for neuronal differentiation after day 7 [35]. Similarly, for the disease-related LCCER data, a marked shift in the network structure occurs on day 4 (Fig 4B), signaling a critical transition of erlotinib-treated PC9 cells towards an irreversible resistance state [39]. The overall dynamic changes of the regulatory network for these datasets are further detailed inS8 Fig. Moreover, a gene cluster comprising high-HNPP or signaling genes (top 5% molecules with the highest local HNPP values) together with low-HNPP genes (top 5% molecules with the lowest local HNPP values), was selected to carry out RNA velocity analysis using expression profiles. In particular, RNA velocities were computed with scVelo [51], which employs a gene-specific kinetic model to estimate cell-state transitions from RNA splicing dynamics. For the pericyte-to-neuron, ICM-to-VEC, and LCCER datasets, as shown in Fig 4C–4E, RNA velocities are projected onto the UMAP embedding as streamlines. These streamline patterns consistently reveal the directional progression from the before-transition to the after-transition phase, thereby uncovering the developmental trends of complex biological processes such as cell differentiation and disease evolution.

### Revealing potential regulatory mechanism of erlotinib-resistance in lung cancer cells

**Fig 5:**
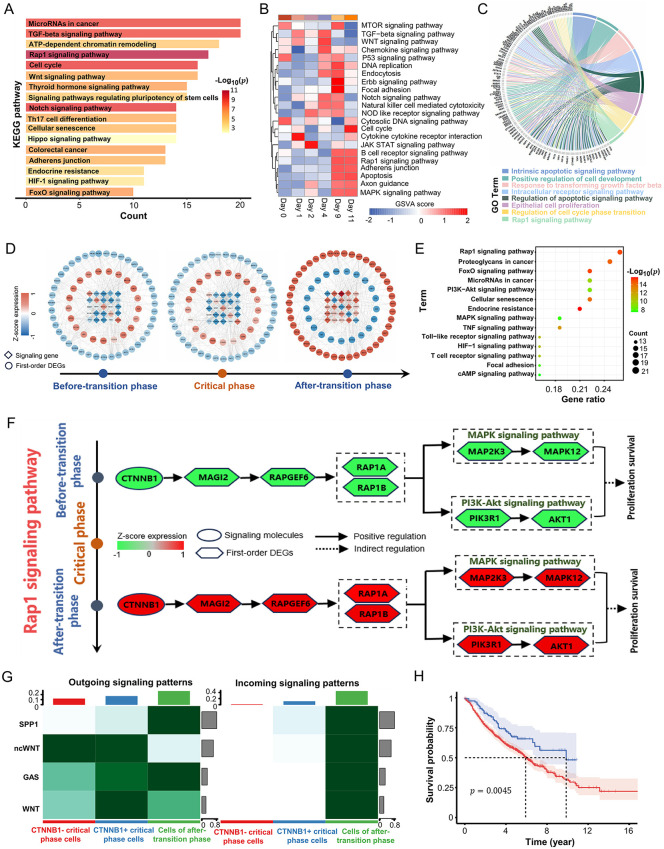
The potential mechanisms underlying erlotinib resistance in lung cancer cells. **(A)** KEGG-based enrichment analysis was conducted to investigate the signaling genes. **(B)** GSVA-based functional assessment reveals that signaling molecules play diverse roles in cancer resistance progression. **(C)** Gene Ontology (GO) analysis suggested that signaling molecules are significantly enriched in biological pathways relevant to tumor resistance. **(D)** Dynamic alterations in the regulatory network formed by signaling molecules and 1st-order DEG neighbors were analyzed during the process of cancer resistance. **(E)** KEGG-based enrichment analysis was performed on the 1st-order DEG neighbors. **(F)** The analysis of signaling molecules and 1st-order DEG neighbors highlighted their involvement in cancer resistance through the Rap1 signaling pathway. **(G)** Cell–cell communication between CTNNB1 + critical-phase cells and after-transition phase cells is mediated via the ncWNT and SPP1 pathways. **(H)** Prognostic significance of CTNNB1 is evaluated in the TCGA-LUAD data.

To reveal insights into the molecular mechanisms of erlotinib-resistance in lung cancer cells, we carried out functional analysis of the signaling genes. Specifically, KEGG pathway enrichment analysis revealed that the signaling genes were significantly enriched in pathways closely associated with tumor erlotinib resistance, including the Rap1 signaling pathway [52], MicroRNAs in cancer [53], and TGF-beta signaling pathway [54] (Fig 5A). Moreover, as shown in Fig 5B, GSVA analysis of signaling genes indicated that the enrichment scores of pathways such as the B cell receptor signaling pathway, Rap1 signaling pathway, Adherens junction, Apoptosis, and MAPK signaling pathway showed an increasing trend during the development of cancer resistance, which may promote erlotinib resistance by enhancing tumor cell proliferation, self-renewal, apoptosis suppression, and adhesion. Additionally, GO enrichment analysis also showed that the signaling molecules are predominantly involved in biological processes associated with tumor resistance, including the intrinsic apoptotic signaling pathway, response to transforming growth factor beta, and regulation of apoptotic signaling pathway (Fig 5C). Additional results of the GO enrichment analysis for cellular components (CC) and molecular functions (MF) are provided in S9 Fig and S9 Text. In addition, we further investigate the functional relevance of signaling genes in embryonic developmental datasets, including the pericyte-to-neuron and hESC-to-DEC datasets (S1-S2 Tables, S10 Fig and S10 Text). To further investigate the potential regulatory mechanisms underlying tumor erlotinib resistance, we analyzed the dynamic behavior of the subnetwork formed by signaling molecules together with their first-order DEG neighbors within the PPI network. Marked alteration of gene expression was observed in the subnetwork before and after the critical phase, implying a major change in regulatory dynamics (Fig 5D). Furthermore, the 1st-order DEG neighbors were found to be enriched in tumor resistance–associated pathways (Fig 5E), including Rap1 signaling, Proteoglycans in cancer, and FoxO signaling pathway.

Our analysis revealed that the Rap1 signaling pathway functions as a major regulatory mechanism underlying cancer resistance. Specifically, upregulation of the signaling gene *CTNNB1* drives the elevated expression of 1st-order DEG neighbor *MAGI2* and *RAPGEF6*, which serve as a membrane-associated scaffold protein and a guanine nucleotide exchange factor (GEF), respectively, and collectively mediate the downstream activation of *RAP1A* and *RAP1B* (Figs 5F and S11). As members of the small GTPase family, *RAP1A* and *RAP1B* are regarded as prerequisites for the initiation of cell survival and proliferative signaling pathways [55]. Activation of *RAP1A* and *RAP1B* amplifies two critical signaling cascades: (i) the *MAP2K3*–*MAPK12* axis, which triggers the MAPK pathway to sustain tumor cell proliferation and survival [56]; and (ii) the *PIK3R1*–*Akt* axis, which maintains PI3K–Akt signaling way to enhance cellular adaptation to drug exposure [57]. The results suggest that the signaling gene *CTNNB1* facilitates acquired resistance in lung cancer cells via the Rap1 pathway, by maintaining pro-survival signaling, inhibiting apoptosis, and reinforcing cell adhesion and motility [58]. To further investigate the role of cell–cell communication in resistance, critical-phase cells were stratified into *CTNNB1*+ and *CTNNB1*- subsets based on *CTNNB1* expression, and receptor–ligand interactions between *CTNNB1* + critical-phase cells and cells of after-transition phase were analyzed to assess their contribution to resistance. The analysis revealed that the *WNT5A*–*FZD2* interaction within the ncWNT pathway [59] promotes the transition of *CTNNB1* + critical-phase cells toward resistant states, while the *SPP1*–*CD44* pair in the SPP1 pathway [60] provides negative feedback regulation, together establishing a positive–negative feedback mechanism that accelerates erlotinib resistance (Figs 5G and S12). Besides, High *CTNNB1* expression was associated with poor prognosis in lung cancer, consistent with the findings (Fig 5H) [61], highlighting its role as a cancer marker of adverse outcomes.

## Discussion

Detecting critical phases in complex biological systems, such as the early stages before tumor onset and key decision points during embryogenesis, provides fundamental insights into biological dynamics. For instance, the identification of critical states before disease worsening can provide timely guidance for clinical intervention and management. The capacity to detect cell fate commitment during embryonic development plays a key role in customizing disease models and performing personalized therapeutic assessments [62]. However, characterizing the dynamics of biological systems and accurately identifying critical phases or tipping points from high-dimensional biological datasets is challenging, as before-transition states often share similarities with critical states in terms of phenotype traits and average molecular expression. Traditional methods struggle with effectiveness and robustness when applied to high-dimensional data with considerable noise, particularly in the case of single-cell expression data. Most existing computational methods focus on the correlations between pairs of molecules (node-to-node interactions) within simple network structures. Integrating higher-order structures into early-warning frameworks has been shown to enhance the reliable characterization of biological processes, as higher-order interactions provide richer insights than pairwise structures [63]. In this paper, we present an innovative approach called HNPP, which combines a modified personalized PageRank model with higher-order structure analysis to identify critical signals in complex biological systems, offering a departure from traditional methods that rely solely on node-to-node interactions within simple network structures. Through the application of the HNPP method to both simulated and six distinct single-cell datasets representing various biological processes, the computational analysis successfully pinpoint their respective critical phases, showcasing the proposed method’s effectiveness in detecting critical signals at the single-cell level.

The novelty and key characteristics of our HNPP method are as follows. Firstly, in contrast to traditional approaches, the HNPP method leverages higher-order structures to offer enhanced insights into molecular multi-body interactions, enabling the accurate and robust identification of critical states from single-cell data and the associated signaling molecules. Secondly, in terms of dynamic change analysis, it performs better in capturing critical transition than other existing critical state detection methods. Thirdly, by assigning a specific importance value (local HNPP score) to each molecule rather than to a group of molecules, it holds significant promise for discovering new network biomarkers and uncovering the potential molecular mechanisms of complex biological processes. Furthermore, our HNPP serves as a data-driven, model-free framework, operating without reliance on model parameter training. Nevertheless, several aspects of this study may benefit from continued exploration. Specifically, as a cell population–based method, HNPP requires an adequate number of cells at each time point, but an excessively large cell size may reduce computational efficiency. Although our method remains tractable for the datasets considered in this study (S3 Table), its computational burden, mainly arising from simplicial-complex construction and the iterative PageRank-like updating procedure, may become more pronounced for very large scRNA-seq atlases with a high gene dimensionality. Consequently, further efforts to improve the scalability of HNPP for large-scale single-cell datasets will be a direction for future work. Moreover, the biological relevance of the identified signaling molecules is supported mainly by indirect evidence, including literature consistency and functional enrichment analysis, and therefore would benefit from more direct validation in future biological applications. For example, perturbation experiments, lineage-tracing analyses, and targeted functional assays in specific biological systems may help further assess the functional roles of the signaling molecules identified by HNPP. Additionally, combining dynamic prediction approaches [64] with our newly proposed framework would be highly valuable for exploring a broader range of complex dynamical systems with intricate higher-order interaction structures.

## Supporting information

S1 Fig Comparison of the dynamic performance of full HNPP, pairwise DNB-based, and HNPP variant models. We analyzed the signal strength of the critical state for the (A)–(C) Pericyte-to-neuron data, (D)–(F) hESC-to-DEC data, and (G)–(I) ICM-to-VEC data.
(TIF)

S2 Fig Under different settings of the adjustable parameter ε, critical signals were observed for (A)–(C) pericyte-to-neuron data and (D)–(F) hESC-to-DEC data. (TIF)

S3 Fig Critical signals under different settings of the adjustable parameter *L.* For the pericyte-to-neuron data, *L* is set as (A) the count of top 3% genes with highest local HNPP, (B) the count of top 5% genes with highest local HNPP, and (C) the count of top 10% genes with highest local HNPP, respectively. Similarly, for the hESC-to-DEC data, *L* is set as (D) the count of top 3% genes with highest local HNPP, (E) the count of top 5% genes with highest local HNPP, and (F) the count of top 10% genes with highest local HNPP, respectively.
(TIF)

S4 Fig A model of an 8-molecule regulatory network. This schematic illustrates a molecular network with 8 nodes, where the dynamic regulatory interactions are described by a stochastic system Eq. (S4). The edges denote regulatory relationships among nodes.
(TIF)

S5 Fig (A)–(B) Pseudotime trajectory illustrating the progression from hepatitis to liver cancer. (C) Proportions of cells derived from hepatitis, cirrhosis, and liver cancer across different cell types.
(TIF)

S6 Fig Critical signals observed in pericyte-to-neuron and hESC-to-DEC datasets under different cell-number settings. For the pericyte-to-neuron data, results are shown for (A) 50% randomly sampled cells, (B) 70% randomly sampled cells, and (C) the full dataset (100% of cells). Similarly, for the hESC-to-DEC data, results are shown for (D) 50% randomly sampled cells, (E) 70% randomly sampled cells, and (F) the full dataset (100% of cells).
(TIF)

S7 Fig Critical signals observed in the pericyte-to-neuron and hESC-to-DEC datasets under different data sparsity thresholds. For the pericyte-to-neuron data, results are shown for (A) 30% zero-expression threshold, (B) 50% zero-expression threshold, and (C) 70% zero-expression threshold. Similarly, for the hESC-to-DEC data, results are shown for (D) 30% zero-expression threshold, (E) 50% zero-expression threshold, and (F) 70% zero-expression threshold.
(TIF)

S8 Fig (A) Temporal dynamics of the regulatory network formed by signaling genes for the pericyte-to-neuron data. (B) Dynamic evolution of the regulatory network composed of signaling genes for the LCCER data.
(TIF)

S9 Fig Gene Ontology (GO) analysis revealed significant enrichment of signaling molecules in biological pathways associated with tumor resistance. (TIF)

S10 Fig Results of KEGG and GO enrichment analyses of the identified signaling genes in the (A)–(B) pericyte-to-neuron data and the (C)–(D) hESC-to-DEC data. The analyses indicate that these signaling genes are enriched in biological processes associated with embryonic development.
(TIF)

S11 Fig Significant differential expression of (A) CTNNB1, MAGI2, and RAPGEF6; (B) RAP1A, RAP1B, and MAP2K3; and (C) PIK3R1, MAPK12, and AKT1 was observed between the pre-critical and post-critical stages of erlotinib resistance in lung cancer cells. (TIF)

S12 Fig Cellular communication between CTNNB1 + critical-phase cells and cells of the after-transition phase. (TIF)

S1 Text Description of main properties of dynamic network biomarker (DNB). (DOCX)

S2 Text Analysis of the effects of major components in HNPP. (DOCX)

S3 Text Signal curve under different values of parameter ϵ. (DOCX)

S4 Text Description of assessing the critical phase. (DOCX)

S5 Text Description of real-world single-cell data from various biological processes. (DOCX)

S6 Text Overview of dynamic systems for simulation data. (DOCX)

S7 Text Constructing a pseudo-temporal trajectory from hepatitis to liver cancer. (DOCX)

S8 Text Performance comparison of various critical detection methods. (DOCX)

S9 Text Functional analysis of the lung cancer cells erlotinib-resistance data. (DOCX)

S10 Text Functional analysis of pericyte-to-neuron and hESC-to-DEC datasets. (DOCX)

S1 Table Information of some important signaling genes in pericyte-to-neuron data. (XLSX)

S2 Table Information of some important signaling genes in hESC-to-DEC data. (XLSX)

S3 Table Runtime comparison of several critical state detection methods. (XLSX)
