# Supplementary figures and images for: HNPP: Higher-order network-based personalized PageRank for detecting critical phase in complex biological systems
Source: PLoS Comput Biol. 2026 Jul 17;22(7):e1014475. doi: 10.1371/journal.pcbi.1014475 (PMC13379042; doi:10.1371/journal.pcbi.1014475)

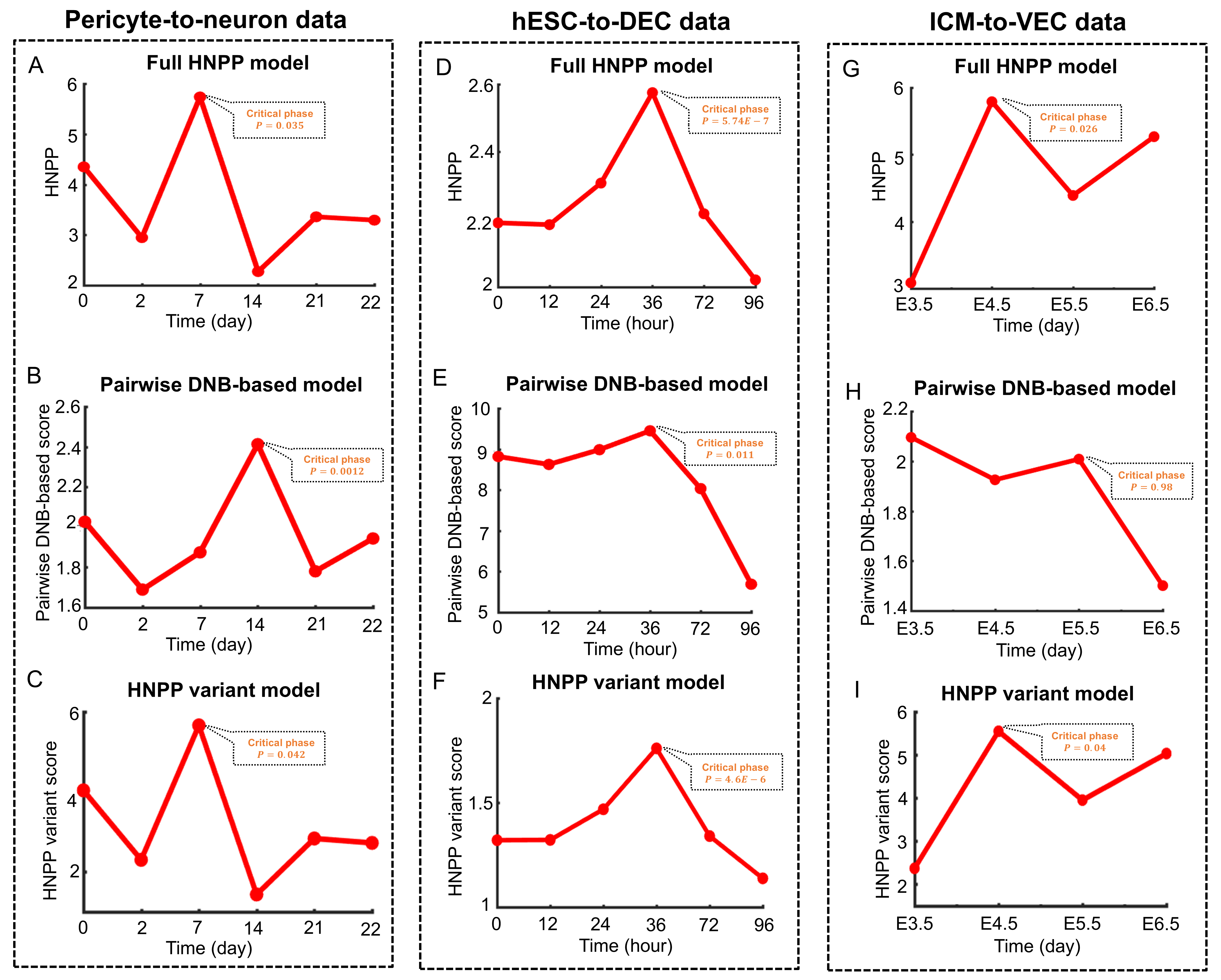

Supplement: S1 Fig — We analyzed the signal strength of the critical state for the (A)–(C) Pericyte-to-neuron data, (D)–(F) hESC-to-DEC data, and (G)–(I) ICM-to-VEC data. (TIF) [file pcbi.1014475.s001.tif]

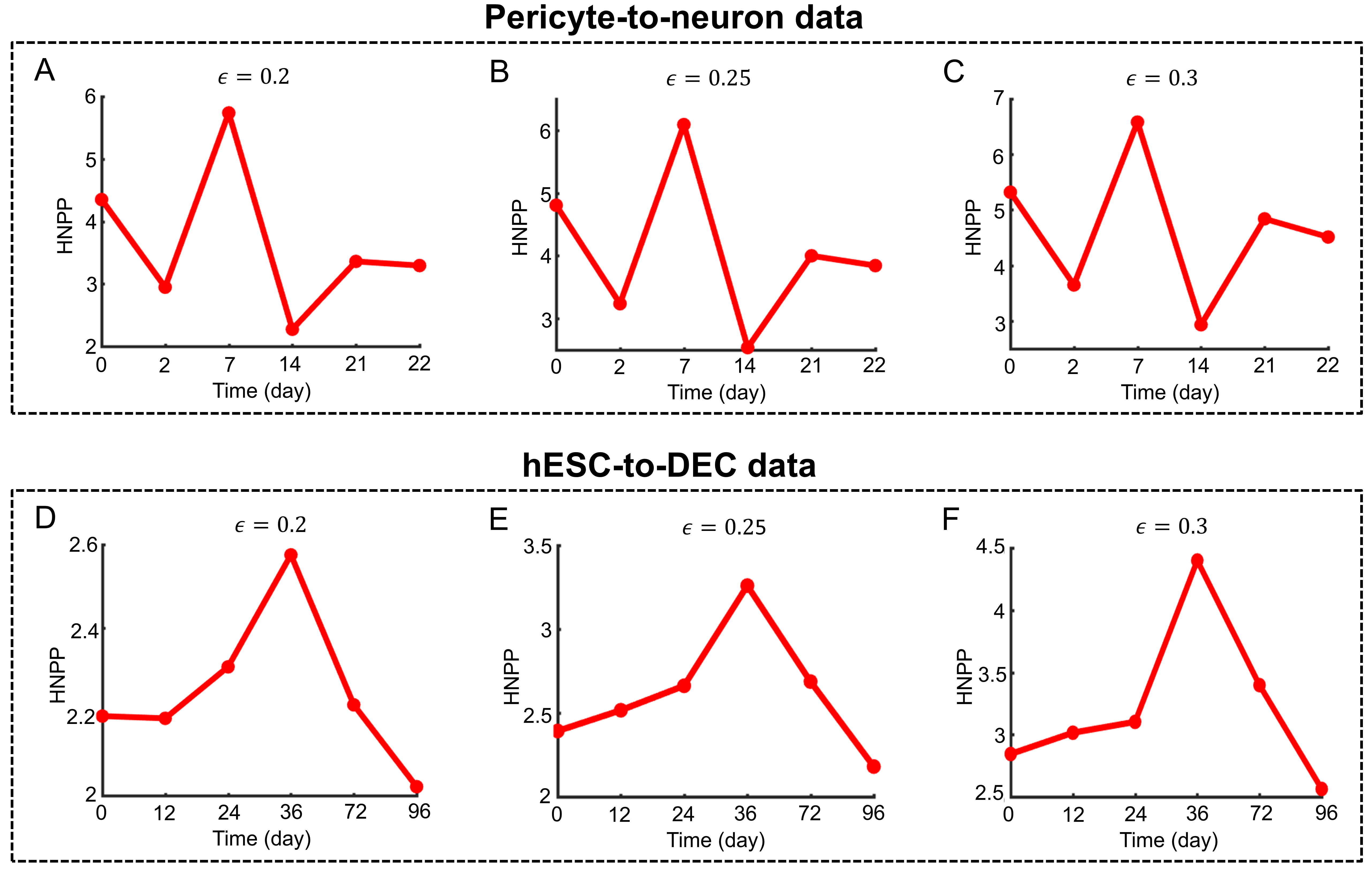

Supplement: S2 Fig — (TIF) [file pcbi.1014475.s002.tif]

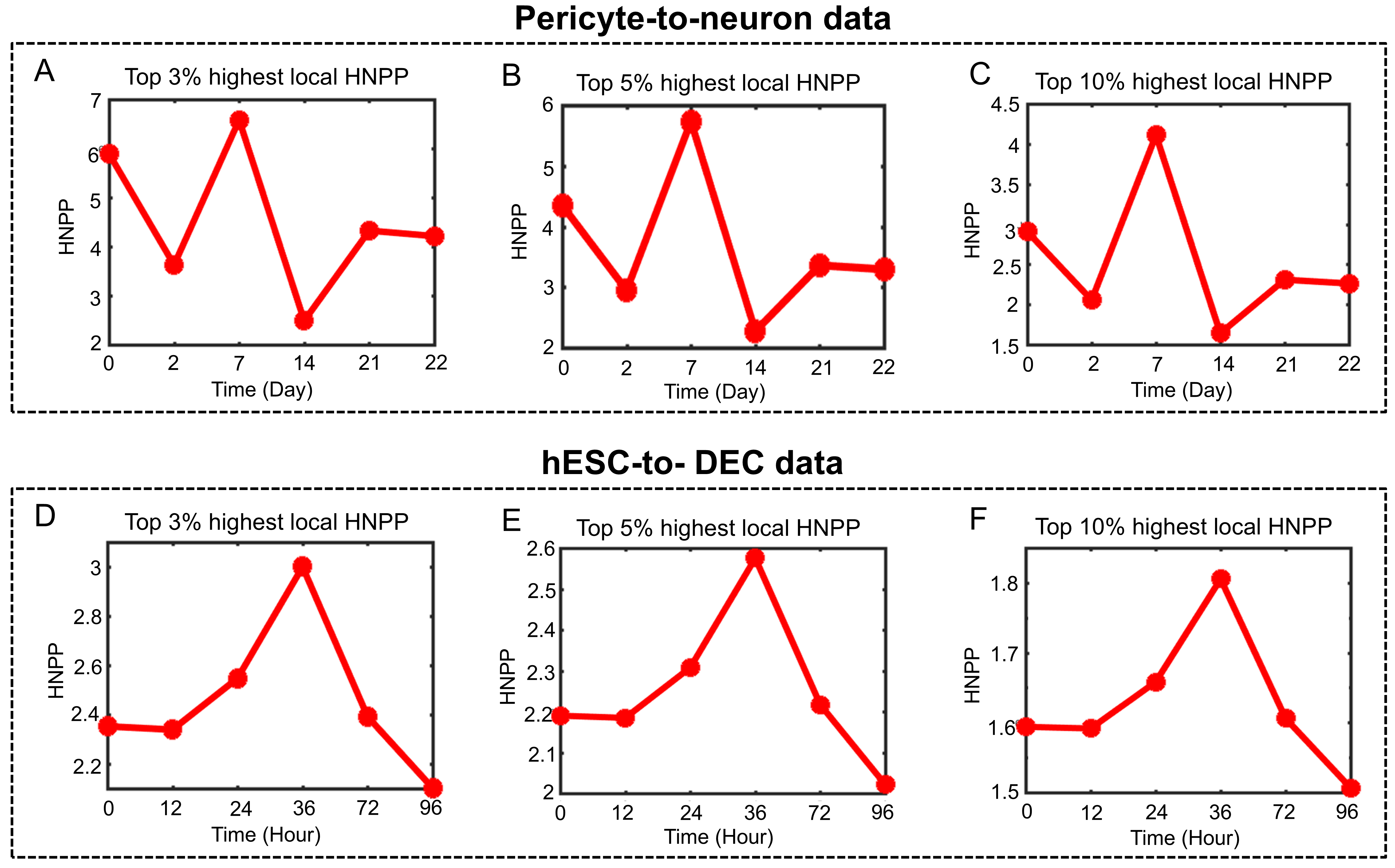

Supplement: S3 Fig — For the pericyte-to-neuron data, L is set as (A) the count of top 3% genes with highest local HNPP, (B) the count of top 5% genes with highest local HNPP, and (C) the count of top 10% genes with highest local HNPP, respectively. Similarly, for the hESC-to-DEC data, L is set as (D) the count of top 3% genes with highest local HNPP, (E) the count of top 5% genes with highest local HNPP, and (F) the count of top 10% genes with highest local HNPP, respectively. (TIF) [file pcbi.1014475.s003.tif]

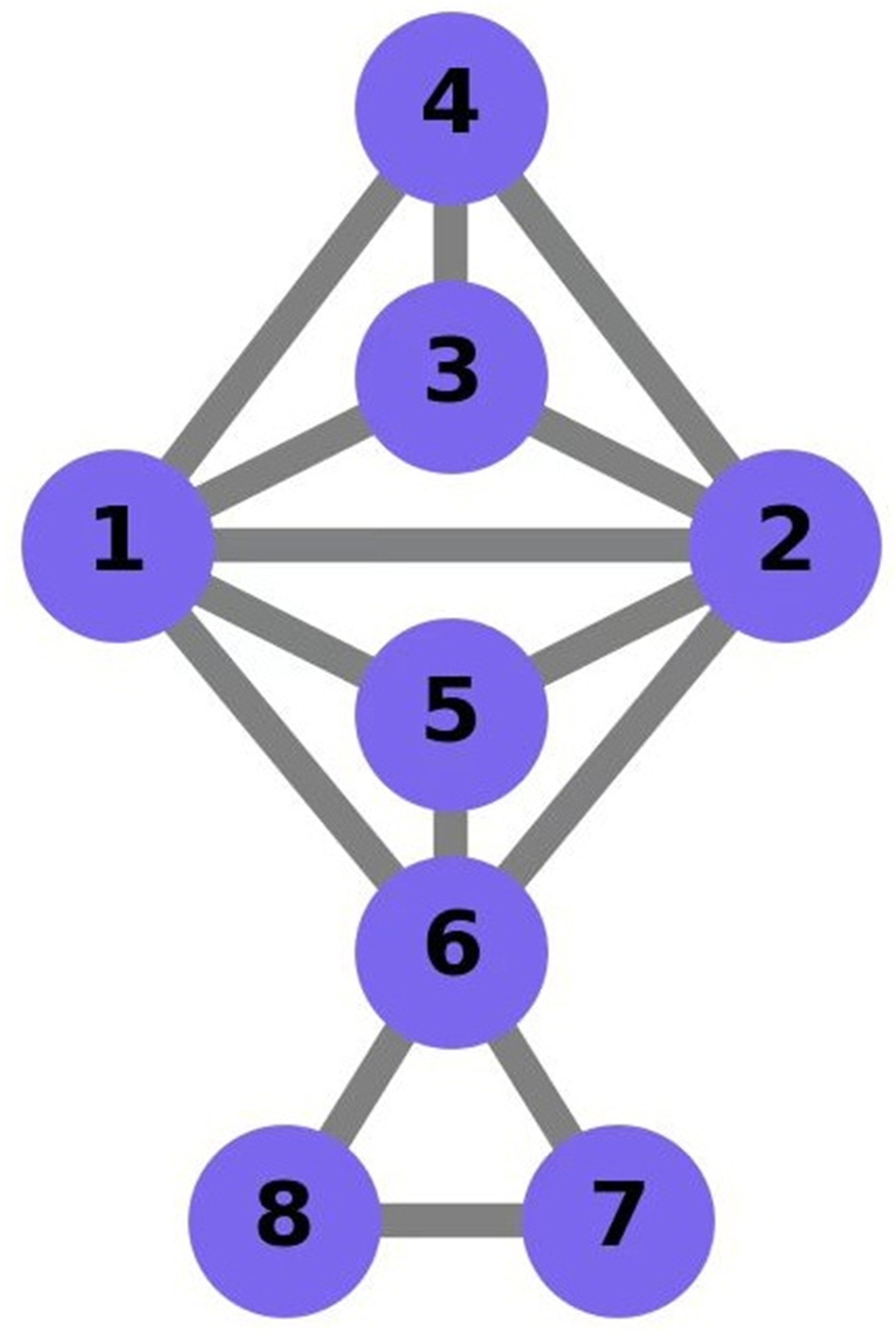

Supplement: S4 Fig — This schematic illustrates a molecular network with 8 nodes, where the dynamic regulatory interactions are described by a stochastic system Eq. (S4). The edges denote regulatory relationships among nodes. (TIF) [file pcbi.1014475.s004.tif]

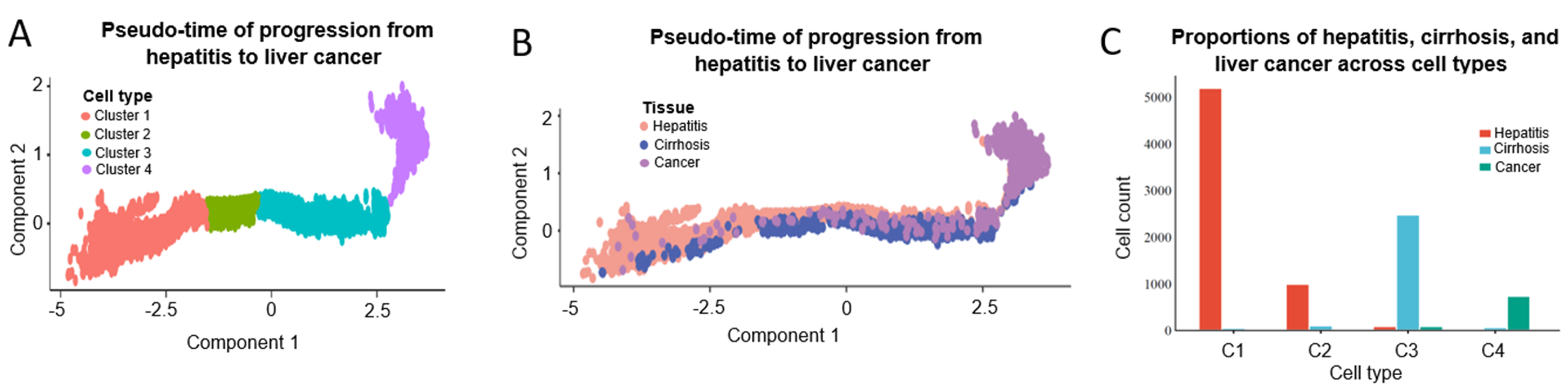

Supplement: S5 Fig — (C) Proportions of cells derived from hepatitis, cirrhosis, and liver cancer across different cell types. (TIF) [file pcbi.1014475.s005.tif]

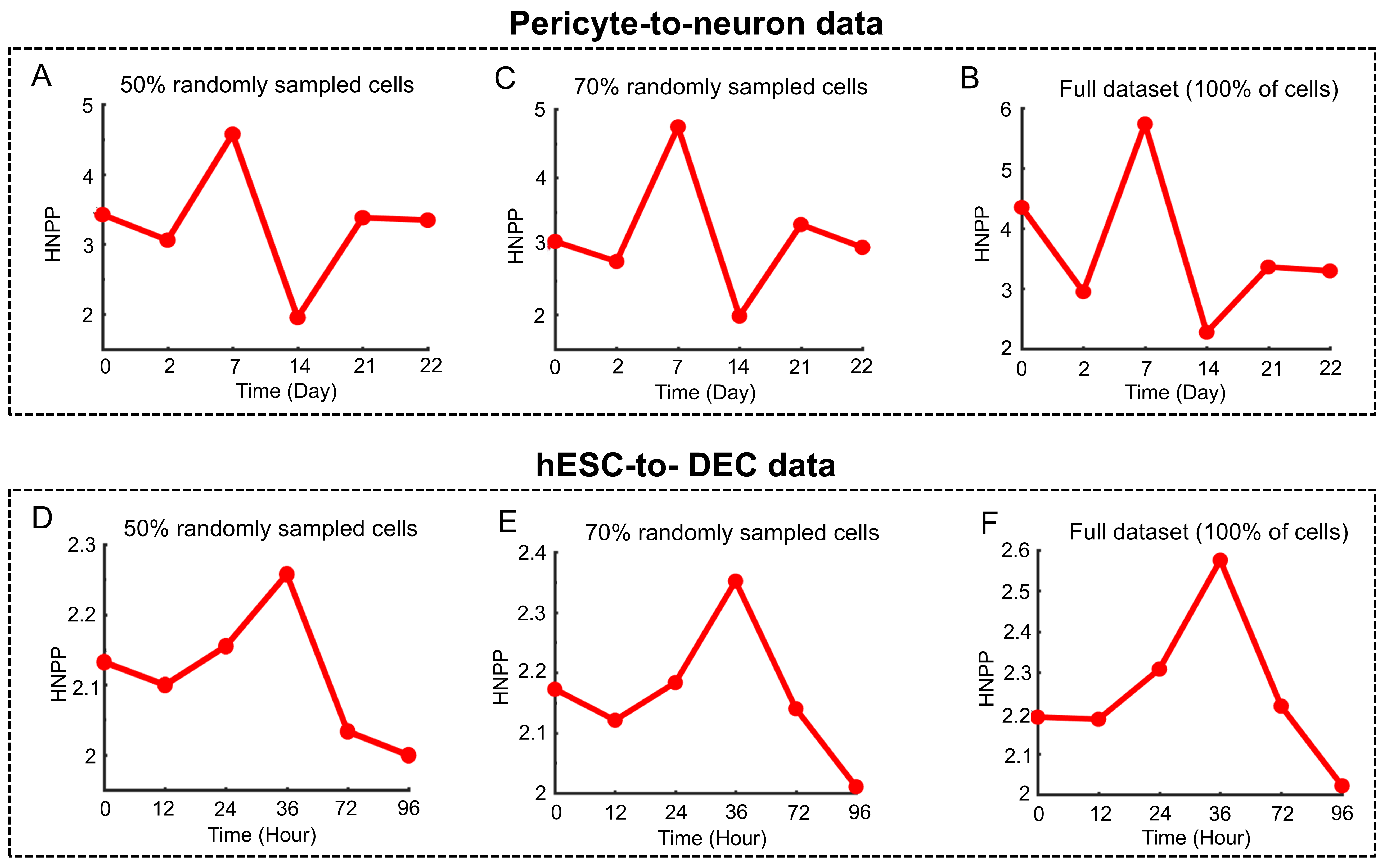

Supplement: S6 Fig — For the pericyte-to-neuron data, results are shown for (A) 50% randomly sampled cells, (B) 70% randomly sampled cells, and (C) the full dataset (100% of cells). Similarly, for the hESC-to-DEC data, results are shown for (D) 50% randomly sampled cells, (E) 70% randomly sampled cells, and (F) the full dataset (100% of cells). (TIF) [file pcbi.1014475.s006.tif]

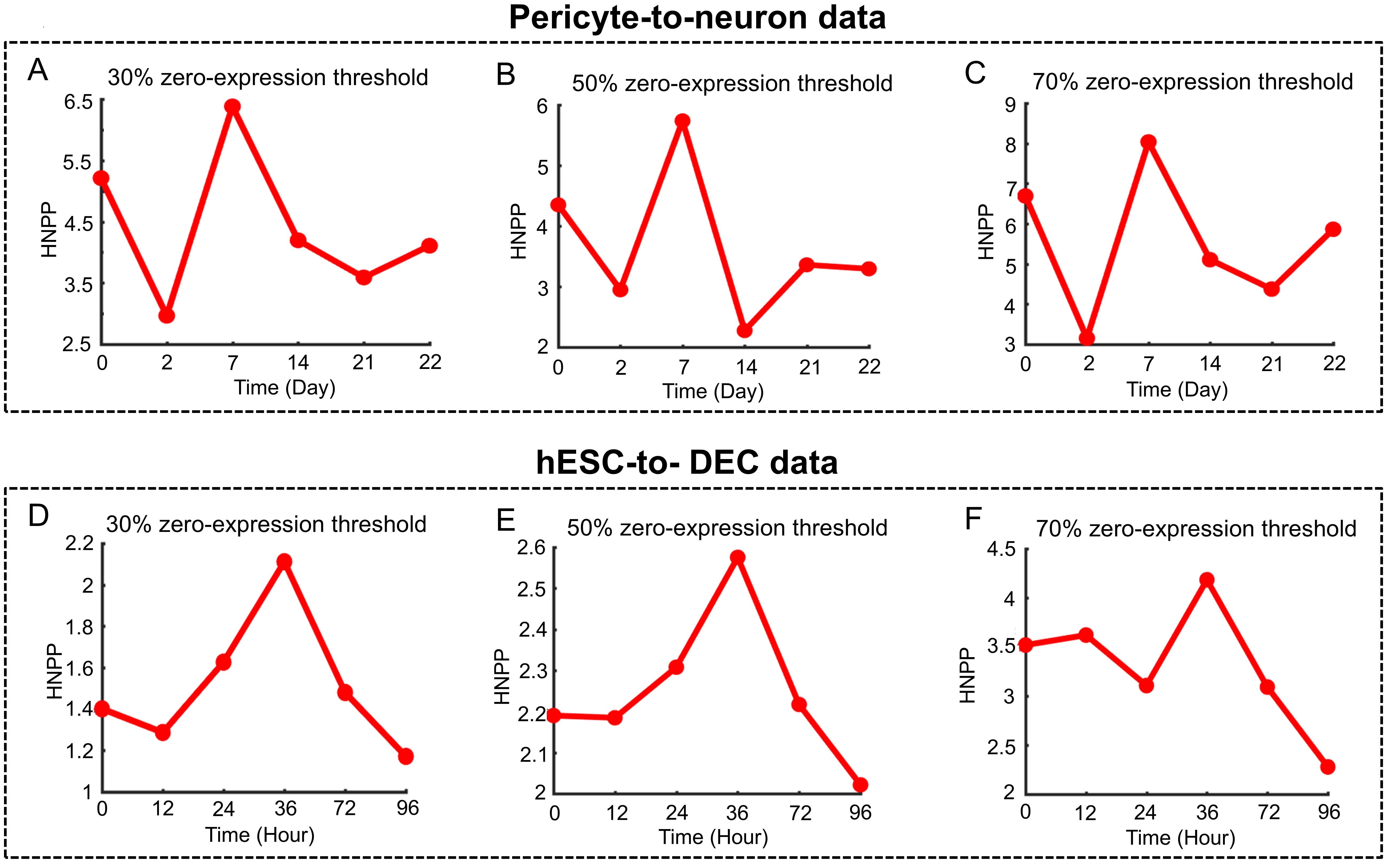

Supplement: S7 Fig — For the pericyte-to-neuron data, results are shown for (A) 30% zero-expression threshold, (B) 50% zero-expression threshold, and (C) 70% zero-expression threshold. Similarly, for the hESC-to-DEC data, results are shown for (D) 30% zero-expression threshold, (E) 50% zero-expression threshold, and (F) 70% zero-expression threshold. (TIF) [file pcbi.1014475.s007.tif]

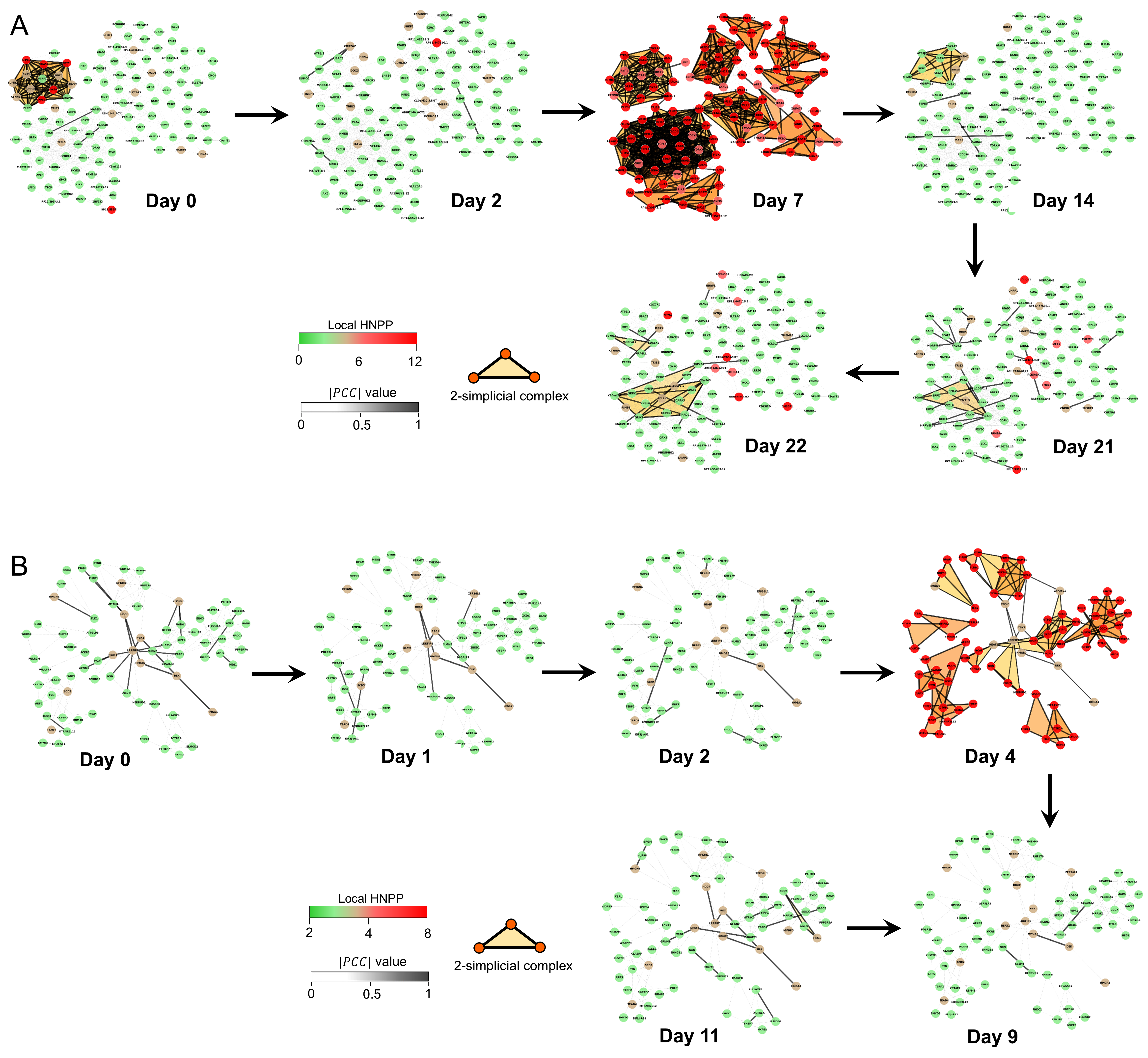

Supplement: S8 Fig — (B) Dynamic evolution of the regulatory network composed of signaling genes for the LCCER data. (TIF) [file pcbi.1014475.s008.tif]

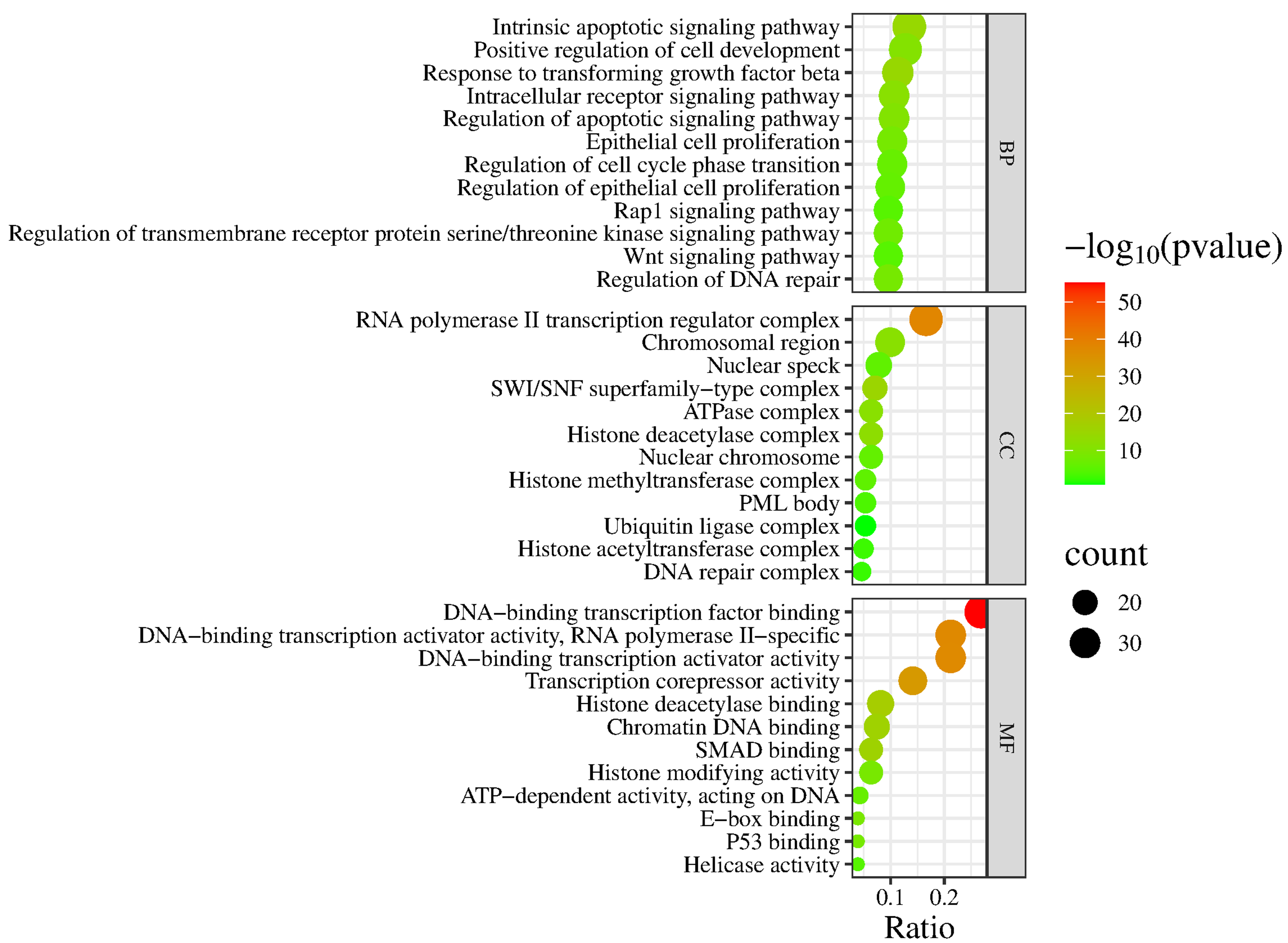

Supplement: S9 Fig — (TIF) [file pcbi.1014475.s009.tif]

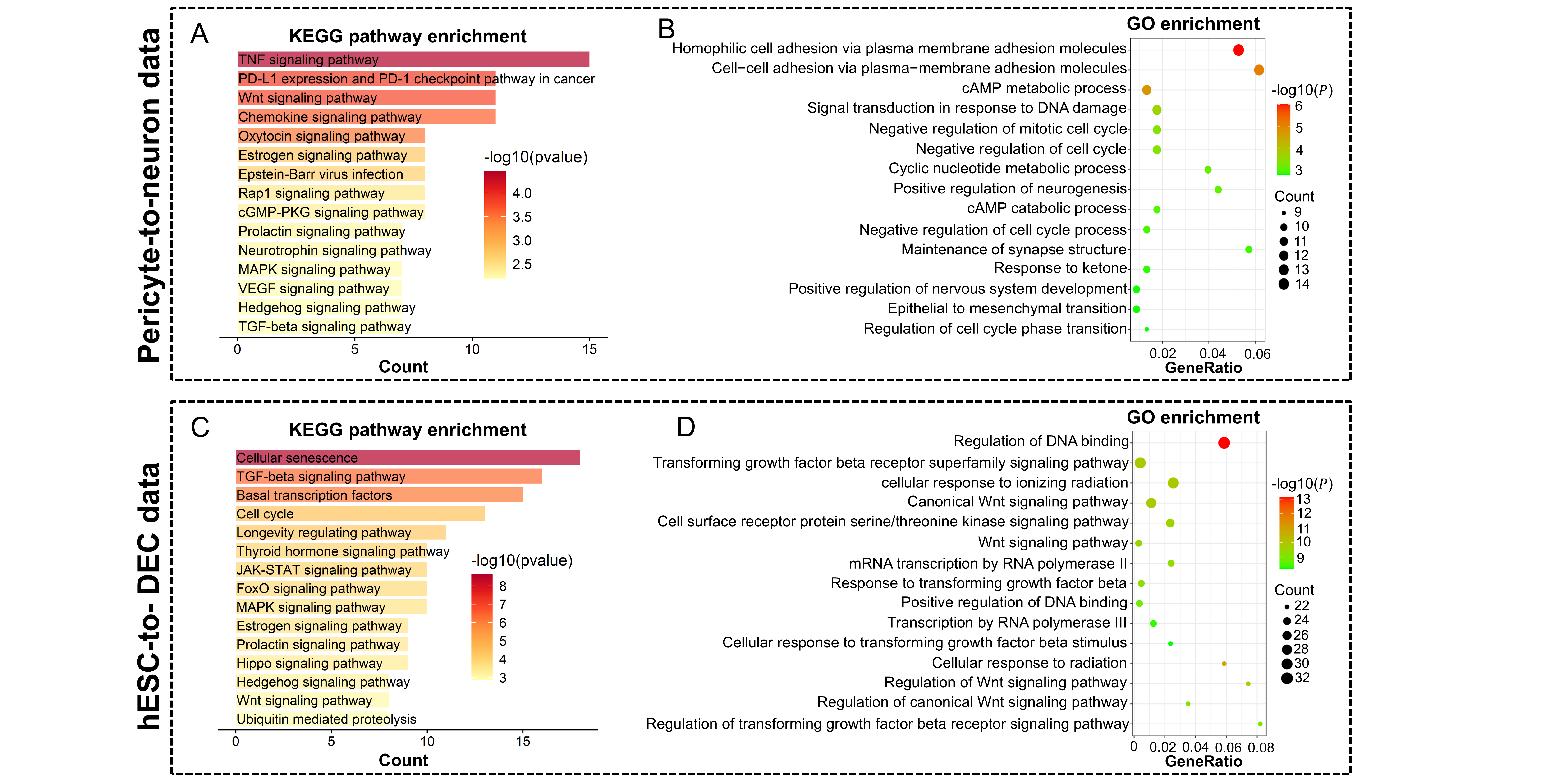

Supplement: S10 Fig — The analyses indicate that these signaling genes are enriched in biological processes associated with embryonic development. (TIF) [file pcbi.1014475.s010.tif]

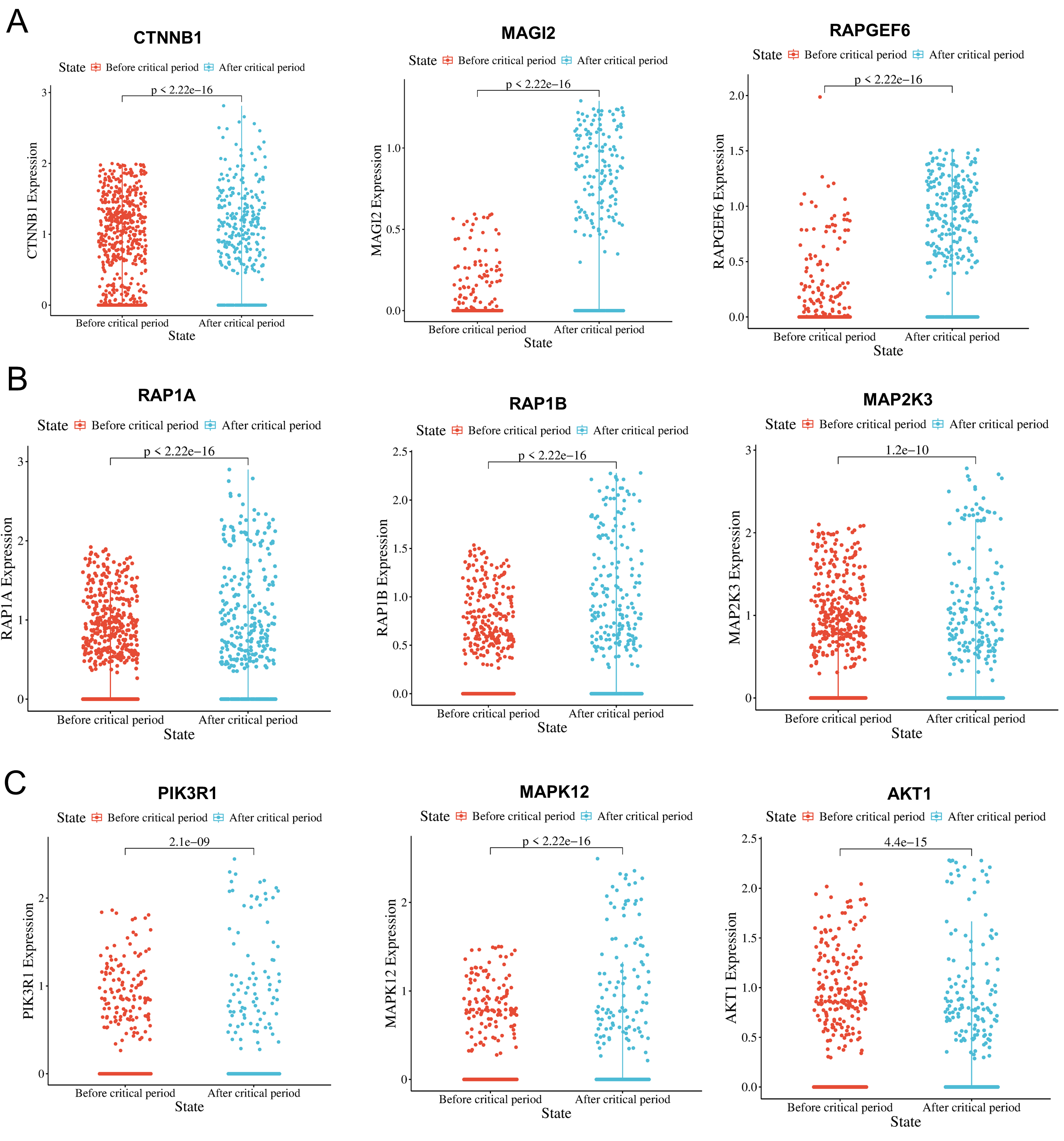

Supplement: S11 Fig — (TIF) [file pcbi.1014475.s011.tif]

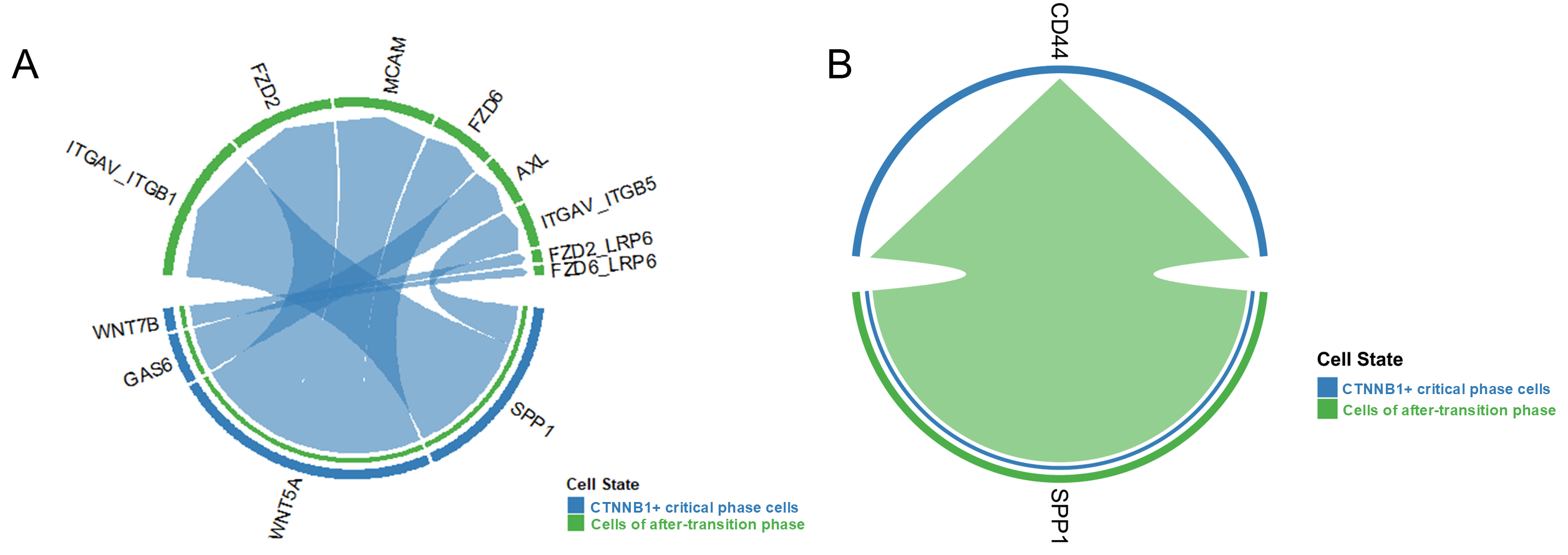

Supplement: S12 Fig — (TIF) [file pcbi.1014475.s012.tif]
