# Supplementary material for: HNPP: Higher-order network-based personalized PageRank for detecting critical phase in complex biological systems
Source: PLoS Comput Biol. 2026 Jul 17;22(7):e1014475. doi: 10.1371/journal.pcbi.1014475 (PMC13379042; doi:10.1371/journal.pcbi.1014475)
Supplement: S1 Text — (DOCX) [file pcbi.1014475.s013.docx]

**Description of main properties of dynamic network biomarker (DNB)**

We present the theoretical basis of the proposed higher-order network-based personalized PageRank (HNPP), rooted in the dynamic network biomarker (DNB) framework [1]. Specifically, the discrete-time dynamical system describing the network evolution can be formulated as follows:

$Z\left( t+1 \right)=f\left( Z\left( t \right);P \right),$  (S1)

where $Z\left( t \right)=(z_{1}\left( t \right),z_{2}\left( t \right),\cdots,z_{n}\left( t \right))$ denotes an 𝑛-dimensional state vector at time step 𝑡, representing the system’s features, whereas $P=(p_{1},p_{2},\cdots,p_{s})$ is the parameter vector that accounts for the slowly varying driving factors. Typically, the mapping$f: R^{n}\times R^{s}\to R^{n}$ is assumed to be nonlinear. For the dynamical evolution function in Eq. (S1) with parameter 𝑃, a bifurcation or critical state occurs when the following conditions hold:

1. $\bar{Z}$ represents a fixed point of the system, defined by the condition $\bar{Z}=f(\bar{Z};P)$.

2. There exists a value $P_{c}$ such that one or a pair of eigenvalues of the Jacobian matrix$\frac{\partial f(Z;P)}{\partial Z}|_{Z=\bar{Z}}$ has modulus equal to 1.

3. When $P\neq P_{c}$, the eigenvalues of the linearized function of $f$ generally do not have a modulus equal to 1.

The combination of the above three conditions and the relevant transverse conditions signifies that the system undergoes a phase transition at $\bar{Z}$, corresponding to a codimension-one bifurcation as $P$ reaches $P_{c}$​. For Eq. (S1), close to $\bar{Z}$ and before $P$ reaches $P_{c}$​, the system is assumed to be at a stable fixed point $\bar{Z}$, with all eigenvalues having modulus between 0 and 1. The parameter $P_{c}$​ corresponding to the state change of the system is referred to as the bifurcation value, also called the critical transition value.

The generic dynamical properties of Eq. (S1) were derived by analyzing the linearized form of Eq. (S1) and the effects of noise perturbations near $\bar{Z}$. Specifically, by defining a new variable $Y(t)=(y_{1}(t),\ldots,y_{n}(t))$ and a transformation matrix $S$, such that $Y(t)=S^{-1}(Z(t)-\bar{Z})$, we obtain:

$Y(t+1)=\Lambda(P)Y(t)+\zeta(t),$ (S2)

Here, $\Lambda(P)$ denotes the diagonalized form of $\frac{\partial f(Z;P)}{\partial Z}|_{Z=\bar{Z}}$, and $\zeta(t)=(\zeta_{1}(t),\ldots,\zeta_{n}(t))$ represents small Gaussian noise terms with zero mean. Let $\sigma_{i}$ represent the small standard deviation of$\zeta_{i}$​ across all $k$. Without any loss of generality, we consider the diagonalized matrix $\Lambda=(\lambda_{1},\ldots,\lambda_{n})$, where each $\lambda_{i}$ falls within the interval (0, 1). Indeed, there are three common scenarios that occur in the diagonalization process; a detailed derivation in the ideal case is provided in the reference [2]. Among the eigenvalues of $\Lambda$, the largest in modulus, denoted $\lambda_{1}$​, approaches 1 as the parameter $P\to P_{c}$ ​. The eigenvalue $\lambda_{1}$​ describes the system's rate of change near a fixed point and is referred to as the dominant eigenvalue. A before-transition state is defined by $\mid\lambda_{1}\mid<1|$, while the critical stage occurs when $\lambda_{1}$​ approaches 1. Without any loss of generality, we consider the first component $y_{1}$​ in$Y$as associated with $\lambda_{1}$​, meaning that $(y_{1},0,\cdots,0)$ serves as the eigenvector of $\lambda_{1}$​. Near a fixed point, it has been shown that a dominant group, known as a dynamic network biomarker (DNB), emerges and satisfies the following conditions as the system approaches a critical point just prior to the transition [1]. The critical properties of the stochastically perturbed linear system described by Eq. (S2) are summarized as follows. Here, $z_{i}$​ in Eq. (S1) represents the value of feature $i$, such as the expression of a gene or protein. When $P$ nears the bifurcation point, following properties hold:

- The variability of each molecule within the DNBs increases sharply;
- The correlations among molecules within the DNBs significantly strengthen;
- The correlations between DNBs and those outside the group weaken.

This result characterizes the generic critical properties of features within the dynamic network biomarker (DNB) at the critical state. DNB properties imply that, near the critical point, a group of fluctuation-prone and tightly correlated biomolecules exhibiting strong cooperative associations mark the forthcoming critical transition. Actually, the qualitative state transition of complex biological system can be detected through analyzing how such dominant variables in molecular associations evolve at the network level. Therefore, our proposed HNPP is designed to capture the criticality of biological systems by incorporating the structural information of higher-order (simplicial) networks into a modified personalized PageRank model, which effectively quantifies quantify dynamic shifts in higher-order interactions to enable more accurate detection.

**References**

[1] Chen L, Liu R, Liu ZP, Li M, Aihara K. Detecting early-warning signals for sudden deterioration of complex diseases by dynamical network biomarkers. Sci Rep. 2012;2:342.

[2] Liu R, Chen P, Aihara K, Chen L. Identifying early-warning signals of critical transitions with strong noise by dynamical network markers. Scientific reports. 2015;5.
