# Supplementary material for: HNPP: Higher-order network-based personalized PageRank for detecting critical phase in complex biological systems
Source: PLoS Comput Biol. 2026 Jul 17;22(7):e1014475. doi: 10.1371/journal.pcbi.1014475 (PMC13379042; doi:10.1371/journal.pcbi.1014475)
Supplement: S2 Text — (DOCX) [file pcbi.1014475.s014.docx]

**Analysis of the effects of major components in HNPP**

To disentangle the effects of the major components of our proposed HNPP, we compare: (i) a pairwise DNB-based scores computed on the same gene sets but without simplicial (higher-order) structures; (ii) an HNPP variant that retains the simplicial (higher-order) component but excludes the personalized PageRank modification (i.e., without the TF-based vector$\vec{\mu}$); and (iii) the full HNPP model as proposed. As shown in Figure S1, the higher-order simplicial construction yields a clear improvement over the standard pairwise DNB formulation. Specifically, as illustrated in Figure S1A–C, for the Pericyte-to-neuron data, the full HNPP model provides a stronger critical signal ($P=0.035$) than the HNPP variant model ($P=0.042$), and both detect earlier warning signals than the pairwise DNB-based model. In the hESC-to-DEC data, it is seen from Figure S1D–F that the full HNPP model ($P=5.74E-7$) shows more statistically significant signal than either the HNPP variant ($P=4.6E-6$) or pairwise DNB-based method ($P=0.11$). When applied to the ICM-to-VEC data, as presented in Figure S1G–I, the signal strength provided by the full HNPP ($P =0.026$) is greater than that of the HNPP variant ($P =0.04$), whereas the pairwise DNB model fails to detect a meaningful critical signal ($P =0.98$). These results indicate that the higher-order simplicial construction makes an independent contribution to the observed performance gains, while personalized PageRank modification with the TF-based vector$\vec{\mu}$ offer further complementary improvements. Overall, the improved performance of HNPP arises from the joint contribution of these components, with the higher-order structure playing a central role in the improvement.
