# Supplementary material for: HNPP: Higher-order network-based personalized PageRank for detecting critical phase in complex biological systems
Source: PLoS Comput Biol. 2026 Jul 17;22(7):e1014475. doi: 10.1371/journal.pcbi.1014475 (PMC13379042; doi:10.1371/journal.pcbi.1014475)
Supplement: S3 Text — (DOCX) [file pcbi.1014475.s015.docx]

**Signal curve under different values of parameter** $\boldsymbol{\epsilon}$

We evaluated whether each set of three genes (gene triplets) could be connected to form a triangle (2-simplex) according to Equations (3) and (7) in the main text, with the adjustable parameter $\epsilon$ primarily set to 0.2. To further evaluate the stability of our method, we analyzed the pericyte-to-neuron and hESC-to-DEC datasets using different values of the parameter $\epsilon$, ranging from the 0.2 to 0.3, to assess critical signals. As illustrated in Figure S2, the HNPP index consistently identified the critical transition point across all tested values of 𝜖, and the overall shape and trend of the signal curve remained essentially unchanged. These results indicate that parameter $\epsilon$ within this range (typically from the 0.2 to 0.3) do not alter the overall trend of the signal curve, demonstrating the robustness of HNPP against the variation of parameter $\epsilon$. Therefore, the stability of the HNPP index with respect to the parameter 𝜖 is well supported, confirming that the method is effectively for detecting early-warning signals across a reasonable range of 𝜖 values.
