# Supplementary material for: HNPP: Higher-order network-based personalized PageRank for detecting critical phase in complex biological systems
Source: PLoS Comput Biol. 2026 Jul 17;22(7):e1014475. doi: 10.1371/journal.pcbi.1014475 (PMC13379042; doi:10.1371/journal.pcbi.1014475)
Supplement: S4 Text — (DOCX) [file pcbi.1014475.s016.docx]

**Description of assessing the critical phase**

To assess the effectiveness of the HNPP score in capturing critical behavior, we apply a one-sample test to examine potential statistical differences between the pre-transition and critical stages. Specifically, the test statistic$ST$ can be found below Eq. (S3) and is used to assess whether the constant $z$ significantly departs from the mean of the vector$\vec{Z}=$($z_{1}$, $z_{2}$, $\cdots$,$z_{n}$):

$ST=\sqrt{n}\frac{\mathrm{mean}(\vec{Z})- z}{\mathrm{SD}(\vec{Z})},$  (S3)

where the notation $\mathrm{mean}\left( \vec{Z} \right)$ stands for the average of vector $\vec{Z}$, whereas $\mathrm{SD}\left( \vec{Z} \right)$ signifies its standard deviation. The $p$-value associated with the index is employed to assess the statistical difference between $\mathrm{mean}\left( \vec{Z} \right)$ and $z$. If the $p$-value is less than or equal to 0.05 ($p$-value$\leq0.05$), it indicates a significant statistical difference between $\mathrm{mean}\left( \vec{Z} \right)$ and $z$. In our research, a time point $t=T>2$is classified as pre- critical phase or critical point when the HNPP score meets the following two conditions: ($i$)$HNPP(T)>HNPP(T-1)$ and ($ii$) $HNPP(T)$ shows a statistically significant difference ($p$-value$\leq0.05$) compared to the average of the vector $HP$=$(HNPP(t=1),HNPP(t=2),\cdots,HNPP(t=T-1))$. When the $HNPP(t=2)$ score is considered indicative of a critical phase when it satisfies two requirements: (i) $HNPP(t=2)$>$HNPP(t=1)$and (ii) $HNPP(t=2)$ is statistically different ($p$-value$\leq0.05$) from the average of the vector$HP$=$(HNPP(t=1),HNPP(t=3))$, the time point $t=2$ is regarded as the critical phase.
