# Supplementary material for: HNPP: Higher-order network-based personalized PageRank for detecting critical phase in complex biological systems
Source: PLoS Comput Biol. 2026 Jul 17;22(7):e1014475. doi: 10.1371/journal.pcbi.1014475 (PMC13379042; doi:10.1371/journal.pcbi.1014475)
Supplement: S5 Text — (DOCX) [file pcbi.1014475.s017.docx]

**Description of real-world single-cell data from various biological processes**

The performance of the HNPP method was evaluated using six real-world single-cell datasets: including embryonic developmental processes such as pericyte-to-neuron reprogramming [1] (GEO: GSE113036), differentiation of human embryonic stem cells (hESC) into definitive endoderm cells (DEC) [2] (GEO: GSE75748), the transition from inner cell mass (ICM) to visceral endoderm cells (VEC) [3] (GEO: GSE100597), and human retinal pigment epithelium (HRPE) development [4] (GEO: GSE107618), as well as complex disease-related phenomena like lung cancer cells erlotinib-resistance (LCCER) [5] (GEO: GSE149383) and hepatitis-to-liver-cancer (HELC) progression [6] (PMID: 36221095). Datasets were obtained from the Gene Expression Omnibus (GEO) database (http://www.ncbi.nlm.nih.gov/geo/). Detailed information regarding the datasets and their sources is provided below.

The pericyte-to-neuron dataset was derived from the direct reprogramming of adult human brain pericytes into induced neuronal cells. Single-cell profiles were collected at multiple time points, including day 0 (76 cells), day 2 (86 cells), day 7 (48 cells), day 14 (283 cells), day 21 (61 cells), and day 22 (69 cells). The processed data are available in the GEO database under accession number GSE113036.

The hESC-to-DEC dataset characterizes the differentiation trajectory of human embryonic stem cells (hESC) from the pluripotent state, through a mesendodermal intermediate, to definitive endoderm cells (DEC). In total, 758 single cells were profiled across six time points by scRNA-seq: 0 h (92 cells), 12 h (102 cells), 24 h (66 cells), 36 h (172 cells), 72 h (138 cells), and 96 h (188 cells). The dataset is publicly accessible in the GEO repository under accession number GSE75748.

The ICM-to-VEC dataset illustrates the differentiation of inner cell mass (ICM) into visceral endoderm cells (VEC). A total of 721 single cells were analyzed by scRNA-seq at four developmental time points: E3.5 (99 cells), E4.5 (105 cells), E5.5 (267 cells), and E6.5 (250 cells). The dataset is available in the GEO repository under accession number GSE100597.

The HRPE dataset provides a single-cell transcriptomic landscape of human retinal pigment epithelium (HRPE) development. A total of 1,683 cells were analyzed by scRNA-seq at ten time points spanning early to late developmental stages: 5w (48 cells), 6w (144 cells), 7w (199 cells), 8w (347 cells), 9w (175 cells), 11w (100 cells), 13w (190 cells), 17w (184 cells), 23w (196 cells), and 24w (100 cells). This dataset is publicly available in the GEO database under accession number GSE107618.

The LCCER dataset describes the development of erlotinib resistance in the non-small cell lung carcinoma (NSCLC) cell line PC9, as reported by Benevolenskaya’s research team. Single-cell gene expression profiles were collected at six time points: Day 0 (756 cells), Day 1 (234 cells), Day 2 (144 cells), Day 4 (99 cells), Day 9 (228 cells), and Day 11 (143 cells). The dataset is publicly available in the GEO repository under accession number GSE149383.

The HELC dataset, representing the progression from hepatitis to liver cancer, was processed using Seurat pipelines. A total of 9,858 cells from hepatitis, cirrhosis, and cancer samples were used to construct the pseudo-time trajectory of hepatitis-to-liver-cancer (HELC) via Monocle2. The progression of HELC were grouped into four clusters: cluster 1 (5,258 cells), cluster 2 (1,109 cells), cluster 3 (2,654 cells), and cluster 4 (837 cells). Clusters 1 and 2 were predominantly hepatitis-derived, while clusters 3 and 4 mainly contained cirrhosis- and cancer-derived cells, respectively, reflecting the progression toward liver cancer. Gene expression data are available in the related publication (PMID: 36221095).

**References**

[1] Karow M, Camp JG, Falk S, Gerber T, Pataskar A, Gac-Santel M, Kageyama J, Brazovskaja A, Garding A, Fan W, Riedemann T, Casamassa A, Smiyakin A, Schichor C, Götz M, Tiwari VK, Treutlein B, Berninger B. Direct pericyte-to-neuron reprogramming via unfolding of a neural stem cell-like program. Nat Neurosci. 2018; 21(7):932-940.

[2] Chu L.F. et al (2016) Single-cell RNA-seq reveals novel regulators of human embryonic stem cell differentiation to definitive endoderm. Genome Biol., 17, 1–20.

[3] Mohammed H, Hernando-Herraez I, Savino A, et al. Single-cell landscape of transcriptional heterogeneity and cell fate decisions during mouse early gastrulation. Cell Rep 2017;20(5):1215–28.

[4] Hu Y, Wang X, Hu B, Mao Y et al. Dissecting the transcriptome landscape of the human fetal neural retina and retinal pigment epithelium by single-cell RNA-seq analysis. PLoS Biol 2019 Jul;17(7):e3000365.

[5] Aissa AF, Islam ABMMK, Ariss MM, Go CC, Rader AE, Conrardy RD, Gajda AM, Rubio-Perez C, Valyi-Nagy K, Pasquinelli M, Feldman LE, Green SJ, Lopez-Bigas N, Frolov MV, Benevolenskaya EV. Single-cel transcriptional changes associated with drug tolerance and response to combination therapies in cancer. Nat Commun. 2021 Mar 12;12(1):1628.

[6] Mo Z, Liu D, Chen Y, Luo J, Li W, Liu J, Yu L, Huang B, Zhang S. Single-cell transcriptomics reveals the role of Macrophage-Naïve CD4 + T cell interaction in the immunosuppressive microenvironment of primary liver carcinoma. J Transl Med. 2022 Oct 11;20(1):466.
