# Supplementary material for: HNPP: Higher-order network-based personalized PageRank for detecting critical phase in complex biological systems
Source: PLoS Comput Biol. 2026 Jul 17;22(7):e1014475. doi: 10.1371/journal.pcbi.1014475 (PMC13379042; doi:10.1371/journal.pcbi.1014475)
Supplement: S6 Text — (DOCX) [file pcbi.1014475.s018.docx]

**Overview of dynamic systems for simulation data**

To showcase the effectiveness of our HNPP, we performed a numerical simulation using an 8-node regulatory network, as depicted in Figure S4. This network is described by a system of stochastic differential equations based on Michaelis-Menten or Hill dynamics, which are widely applied in the analysis of gene regulation. These dynamics encompass key biological processes such as transcription, translation, diffusion, and translocation, all of which impact gene expression [1,2]. The following 8 differential equations model the regulation of 8 genes within the network, with gene regulation represented by Michaelis-Menten kinetics, except for the degradation rates, which are assumed to be linearly proportional to gene concentrations. The specific dynamics of this 8-node network are captured by the following set of equations.

$\left\{\begin{matrix} \frac{\text{d}z_{1}(t)}{dt}=\frac{(8-4|p|)z_{2}(t)}{15(1+z_{2}(t))}-\frac{(4+4|p|)}{15}z_{1}(t)+\zeta_{1}(t) \\ \frac{\text{d}z_{2}(t)}{dt}=\frac{(4-2|p|)z_{1}(t)}{15(1+z_{1}(t))}-\frac{(8+2|p|)z_{2}(t)}{15}+\zeta_{2}(t) \\ \frac{\text{d}z_{3}(t)}{dt}=\frac{(4|p|-10)}{15}+\frac{(5-2|p|)}{15(1+z_{1}(t))}+\frac{(5-2|p|)}{15(1+z_{2}(t))}-z_{3}(t)+\frac{1}{5}z_{4}(t)+\zeta_{3}(t) \\ \frac{\text{d}z_{4}(t)}{dt}=\frac{(4|p|-12)}{15}+\frac{(6-2|p|)z_{1}(t)}{15(1+z_{1}(t))}+\frac{(6-2|p|)z_{2}(t)}{15(1+z_{2}(t))}-\frac{6}{5}z_{4}(t)+\zeta_{4}(t) \\ \frac{\text{d}z_{5}(t)}{dt}=\frac{(4|p|-14)}{15}+\frac{(7-2|p|)z_{1}(t)}{15(1+z_{1}(t))}+\frac{(7-2|p|)z_{2}(t)}{15(1+z_{2}(t))}-\frac{7}{5}z_{5}(t)+\zeta_{5}(t) \\ \frac{\text{d}z_{6}(t)}{dt}=-\frac{11}{15}+\frac{1}{15(1+z_{1}(t))}+\frac{1}{15(1+z_{2}(t))}+\frac{z_{3}(t)}{5(1+z_{3}(t))}+\frac{1}{5(1+z_{5}(t))}+\frac{1}{5(1+z_{7}(t))} \\ +\frac{1}{5(1+z_{8}(t))}-\frac{8}{5}z_{6}(t)+\zeta_{6}(t) \\ \frac{\text{d}z_{7}(t)}{dt}=\frac{z_{8}(t)}{10(1+z_{8}(t))}-\frac{19}{10}z_{7}(t)+\zeta_{7}(t) \\ \frac{\text{d}z_{8}(t)}{dt}=\frac{z_{7}(t)}{10(1+z_{7}(t))}-\frac{19}{10}z_{8}(t)+\zeta_{8}(t) \end{matrix}\right.$(S4)

where $p$ is a scalar control parameter, and $\zeta_{i}(t)$ (*i* = 1, 2, $\cdots$, 8) represent Gaussian noises with zero mean and covariance $k_{ij}=\mathrm{Cov}(\zeta_{i},\zeta_{j})$. the concentrations of mRNA-$i$ are described by $z_{i}(t)$ (*i* = 1, 2, $\cdots$, 8). In Eq.(S4), the degradation rates of the mRNAs are given by: $R\text{=}\left(\frac{(4+4|p|)}{15}\text{,}\frac{(8+2|p|)}{15}\text{,1,}\frac{6}{5}\text{,}\frac{7}{5}\text{,}\frac{8}{5}\text{,}\frac{19}{10}\text{,}\frac{19}{10}\right).$ $\bar{Z}=(\bar{z}_{1},\bar{z}_{2},\ldots,\bar{z}_{8})=(0,0,\ldots,0)$ represents the stable equilibrium point of the dynamic system outlined in Eq. (S4). By applying the Euler method, Eq. (S4) can be transformed into a corresponding set of discrete equations using a small time step of$\Delta t$.

$\left\{\begin{matrix} \begin{matrix} z_{1}(k+1) \end{matrix}=z_{1}(k)+\left[\frac{(8-4|p|)z_{2}(k)}{15(1+z_{2}(k))}-\frac{(4+4|p|)}{15}z_{1}(k)+\zeta_{1}(k)\right]\Delta t \\ \begin{matrix} z_{2}(k+1) \end{matrix}=z_{2}(k)+\left[\frac{(4-2|p|)z_{1}(k)}{15(1+z_{1}(k))}-\frac{(8+2|p|)z_{2}(k)}{15}+\zeta_{2}(k)\right]\Delta t \\ \begin{matrix} z_{3}(k+1) \end{matrix}=z_{3}(k)+\left[\frac{(4|p|-10)}{15}+\frac{(5-2|p|)}{15(1+z_{1}(k))}+\frac{(5-2|p|)}{15(1+z_{2}(k))}-z_{3}(k)+\frac{1}{5}z_{4}(k)+\zeta_{3}(k)\right]\Delta t \\ \begin{matrix} z_{4}(k+1) \end{matrix}=z_{4}(k)+\left[\frac{(4|p|-12)}{15}+\frac{(6-2|p|)z_{1}(k)}{15(1+z_{1}(k))}+\frac{(6-2|p|)z_{2}(k)}{15(1+z_{2}(k))}-\frac{6}{5}z_{4}(k)+\zeta_{4}(k)\right]\Delta t \\ \begin{matrix} z_{5}(k+1) \end{matrix}=z_{5}(k)+\left[\frac{(4|p|-14)}{15}+\frac{(7-2|p|)z_{1}(k)}{15(1+z_{1}(k))}+\frac{(7-2|p|)z_{2}(k)}{15(1+z_{2}(k))}-\frac{7}{5}z_{5}(k)+\zeta_{5}(k)\right]\Delta t \\ \begin{matrix} z_{6}(k+1) \end{matrix}=z_{6}(k)+\left[-\frac{11}{15}+\frac{1}{15(1+z_{1}(k))}+\frac{1}{15(1+z_{2}(k))}+\frac{z_{3}(k)}{5(1+z_{3}(k))}+\frac{1}{5(1+z_{5}(k))}+\frac{1}{5(1+z_{7}(k))}\right. \\ \left.+\frac{1}{5(1+z_{8}(k))}-\frac{8}{5}z_{6}(k)+\zeta_{6}(k)\right]\Delta t \\ \begin{matrix} z_{7}(k+1) \end{matrix}=z_{7}(k)+\left[\frac{z_{8}(k)}{10(1+z_{8}(k))}-\frac{19}{10}z_{7}(k)+\zeta_{7}(k)\right]\Delta t \\ \begin{matrix} z_{8}(k+1) \end{matrix}=z_{8}(k)+\left[\frac{z_{7}(k)}{10(1+z_{7}(k))}-\frac{19}{10}z_{8}(k)+\zeta_{8}(k)\right]\Delta t \end{matrix}\right.$(S5)

Where $Z(k)$ refers to the vector $Z(t)$ at the time instant $k\Delta t$. The Jacobian matrix for Eq. (S5) is indicated as $J=\left.\frac{\partial f(Z(k);p)}{\partial Z}\right|_{Z=\bar{Z}}$, with

$J=e^{\Delta t\bullet A}$. (S6)

with $A$ is the coefficient matrix of the linearized system of Eq.(S4). Setting $\Delta t=1$ enables the derivation of eight distinct eigenvalues from Eq. (S6). The leading eigenvalue satisfies the condition $0.67^{|p|}$→ 1 when $p$→ 0. This behavior indicates that the dominant eigenvalue of the differential system described by Eq. (S5) steadily tends toward 0 from the negative side $p$→ 0. Consequently, the equilibrium point $\bar{Z}$ is considered stable if $p\in(0, 1]$. This specific parameter$p=0$ represents the bifurcation point, signaling a qualitative change in the system's dynamics.

**References**

[1] Becskei, A. & Serrano, L. Engineering stability in gene networks by autoregulation,Nature 405, 590–593(2000).

[2] Chen, L. & Aihara, K. Stability of genetic regulatory networks with time delay, IEEE Trans. Circuits Syst. I 49, 602–608(2002). eterogeneity in colorectal cancer patients using single-cell RNA-seq data [J]. 2022, 146-146.
