# Supplementary material for: HNPP: Higher-order network-based personalized PageRank for detecting critical phase in complex biological systems
Source: PLoS Comput Biol. 2026 Jul 17;22(7):e1014475. doi: 10.1371/journal.pcbi.1014475 (PMC13379042; doi:10.1371/journal.pcbi.1014475)
Supplement: S7 Text — (DOCX) [file pcbi.1014475.s019.docx]

**Constructing a** **pseudo-temporal trajectory from hepatitis to liver cancer**

The HELC dataset, representing the progression from hepatitis to liver cancer, was processed using the Seurat pipeline. We employed a pseudo-time inference strategy using the Monocle algorithm to reconstruct the trajectory of hepatitis-to-liver-cancer (HELC) progression. Specifically, the pseudo-temporal trajectory of HELC was categorized into four distinct clusters: cluster 1 (5,258 cells), cluster 2 (1,109 cells), cluster 3 (2,654 cells), and cluster 4 (837 cells) (Figure S5A). Therefore, for non-time-series single-cell dataset of HELC, the progression of HELC can be categorized into four distinct clusters by constructing a pseudo-temporal trajectory. Clusters 1 and 2 were predominantly hepatitis-derived, while clusters 3 and 4 mainly contained cirrhosis- and cancer-derived cells (Figure S5B-C), respectively, reflecting the progression toward liver cancer.
