# Supplementary material for: HNPP: Higher-order network-based personalized PageRank for detecting critical phase in complex biological systems
Source: PLoS Comput Biol. 2026 Jul 17;22(7):e1014475. doi: 10.1371/journal.pcbi.1014475 (PMC13379042; doi:10.1371/journal.pcbi.1014475)
Supplement: S8 Text — (DOCX) [file pcbi.1014475.s020.docx]

**Performance comparison of** **various critical detection methods**

Many critical detection methods, such as Gaussian graphical optimal transport (GGOT) [1], single-sample landscape entropy (SLE) [2], BioTIP [3], directed-network rank score (DNRS) [4], and module-based dynamic network biomarker (M-DNB) [5], have been applied to assess the criticality of complex diseases. GGOT, SLE, BioTIP, DNRS, and M-DNB are computational methods for critical transition analysis based on dynamic molecular changes and can be viewed as extensions or developments of the DNB framework. Briefly, GGOT characterizes stage-specific molecular states using Gaussian graphical models and quantifies progression between stages through optimal transport, thereby identifying critical transitions from changes in molecular-state distributions. SLE is a tipping-point analysis that uses landscape entropy to detect phase transitions by capturing molecular network-level changes. BioTIP advances tipping-point analysis by integrating molecular fluctuation, correlation structure, and network information to identify significant critical transition signals (CTSs) and their regulatory factors. DNRS is a model-free method that captures dynamic changes in directed gene cooperative effects and uses a rank-based score to detect early-warning signals of critical transitions. M-DNB is a module-based framework that detects tipping points and identifies key DNB factors by quantifying dynamic changes at the network-module level. Therefore, these methods were selected as comparison methods for critical transition analysis. Compared with these approaches, our HNPP detects more effective critical signals and provides earlier warning signals before the known biological transition (Table 1 of main text), thereby demonstrating its enhanced ability to pinpoint critical phases in complex biological processes.

**References**

[1] Hua W, Cui R, Yang H, Zhang J, Liu C, Sun J. Uncovering critical transitions and molecule mechanisms in disease progressions using Gaussian graphical optimal transport. Commun Biol. 2025;8(1):575.

[2] Liu R, Chen P, Chen L. Single-sample landscape entropy reveals the imminent phase transition during disease progression. Bioinformatics. 2020;36(5):1522-1532.

[3] Yang XH, Goldstein A, Sun Y, Wang Z, Wei M, Moskowitz IP, Cunningham JM. Detecting critical transition signals from single-cell transcriptomes to infer lineage-determining transcription factors. Nucleic Acids Res. 2022;50(16):e91.

[4] Zhong J, Han C, Wang Y, Chen P, Liu R. Identifying the critical state of complex biological systems by the directed-network rank score method. Bioinformatics. 2022;38(24):5398-5405.

[5] Li L, Xu Y, Yan L, Li X, Li F, Liu Z, Zhang C, Lou Y, Gao D, Cheng X, Chen L. Dynamic network biomarker factors orchestrate cell-fate determination at tipping points during hESC differentiation. Innovation (Camb). 2022;4(1):100364.
