# Supplementary material for: HNPP: Higher-order network-based personalized PageRank for detecting critical phase in complex biological systems
Source: PLoS Comput Biol. 2026 Jul 17;22(7):e1014475. doi: 10.1371/journal.pcbi.1014475 (PMC13379042; doi:10.1371/journal.pcbi.1014475)
Supplement: S9 Text — (DOCX) [file pcbi.1014475.s021.docx]

**Functional analysis of the** **lung cancer cells erlotinib-resistance data**

To reveal insights into the molecular mechanisms of erlotinib-resistance in lung cancer cells, we carried out functional analysis of the signaling molecules. Specifically, the results of the Gene Ontology (GO) enrichment analysis, as shown in Figure S9, reveal that signaling molecules are predominantly implicated in biological processes (BP), cellular components (CC), and molecular functions (MF) associated with tumor resistance, suggesting their critical role in the regulation of acquired resistance in NSCLC. Notably, pathways such as the "Cell cycle," "Regulation of DNA repair," and "Regulation of apoptotic signaling," along with classic resistance-associated pathways including "Wnt" and "TGF-β," were significantly enriched. Additionally, the Rap1 signaling pathway, functioning as a central hub for multiple upstream and downstream effectors, was also notably enriched, implying that signaling molecules may modulate cell adhesion to enhance cell proliferation and survival. Together, these findings underscore the essential role of signaling molecules in governing tumor cell survival, and migration, which are key processes in the development of drug resistance.
