# Supplementary material for: HNPP: Higher-order network-based personalized PageRank for detecting critical phase in complex biological systems
Source: PLoS Comput Biol. 2026 Jul 17;22(7):e1014475. doi: 10.1371/journal.pcbi.1014475 (PMC13379042; doi:10.1371/journal.pcbi.1014475)
Supplement: S10 Text — (DOCX) [file pcbi.1014475.s022.docx]

**Functional analysis of pericyte-to-neuron and hESC-to-DEC datasets**

We evaluated the biological relevance of the identified signaling genes in the embryonic developmental datasets by performing KEGG pathway and Gene Ontology (GO) enrichment analyses, together with supporting evidence from the literature. Specifically, for the pericyte-to-neuron data, KEGG enrichment analysis showed that the identified signaling genes were significantly enriched in pathways such as the TNF signaling pathway, Wnt signaling pathway, and chemokine signaling pathway (Figure S10A), which are closely related to embryonic development. Moreover, GO enrichment analysis indicated that these genes were significantly enriched in cell–cell adhesion via plasma-membrane adhesion molecules, cAMP metabolic process, and signal transduction in response to DNA damage (Figure S10B), suggesting their involvement in early embryonic tissue development. Similarly, in the hESC-to-DEC data, the identified signaling genes were predominantly enriched in cellular senescence, the TGF-beta signaling pathway, and the cell cycle (Figure S10C). GO enrichment analysis further showed enrichment in processes such as regulation of DNA binding, cellular response to ionizing radiation, and the canonical Wnt signaling pathway (Figure S10D), which are also known to play important roles in embryonic development. In addition, for both the pericyte-to-neuron and hESC-to-DEC datasets, several identified signaling genes have been reported in the literature to have important functions in the corresponding developmental processes (Tables S1 and S2), further supporting the developmental relevance of these signaling genes.
